# How Can Gene-Expression Information Improve Prognostic Prediction in TCGA Cancers: An Empirical Comparison Study on Regularization and Mixed Cox Models

**DOI:** 10.3389/fgene.2020.00920

**Published:** 2020-08-21

**Authors:** Xinghao Yu, Ting Wang, Shuiping Huang, Ping Zeng

**Affiliations:** ^1^Department of Epidemiology and Biostatistics, School of Public Health, Xuzhou Medical University, Xuzhou, China; ^2^Center for Medical Statistics and Data Analysis, School of Public Health, Xuzhou Medical University, Xuzhou, China

**Keywords:** linear mixed model, regularization method, Cox model, gene expression, prognostic prediction, the Cancer Genome Atlas

## Abstract

**Background:**

Previous cancer prognostic prediction models often consider only the most important transcriptomic expressions, and their power is limited. It is unknown whether prediction power can be further improved when additional transcriptomic information is incorporated.

**Methods:**

To integrate transcriptomes, four models are compared based on 32 types of cancer in the Cancer Genome Atlas, including the general Cox model with only clinical covariates, the Cox model with a lasso penalty (coxlasso), the Cox model with an elastic net penalty (coxenet), and the mixed-effects Cox model (coxlmm). Furthermore, we partition the survival variance into the relative contribution of clinical and transcriptomic components within the framework of coxlmm. Finally, the influence of different numbers of genes was evaluated in the context of coxlmm.

**Results:**

Compared with the clinical covariates–only Cox model, the average prediction gain was 2.4% for coxlasso, 4.2% for coxenet, and 7.2% for coxlmm across 16 low-censored cancers; a significant elevation of prediction power was observed for SARC, SKCM, LGG, PAAD, and HNSC. Similar findings were observed for all 32 cancers with the average prediction gain of 2.7, 3.8, and 5.8% for coxlasso, coxenet, and coxlmm. Coxlmm always had comparable or better prediction performance relative to coxlasso and coxenet with an average of 2.8% prediction improvement across the 16 low-censored cancers. In addition, it is shown that the predictive accuracy of coxlmm generally increases with the number of genes included. The survival variance partition analysis demonstrates that the transcriptomic contribution was higher for some cancers (e.g., LGG, CESC, PAAD, SKCM, and SARC) and lower for others (e.g., BRCA, COAD, KIRC, and STAD).

**Conclusion:**

This study demonstrates that the integration of transcriptomic information can substantially improve prognostic prediction accuracy, but the prediction performance is cancer-specific and varies across cancer types. It further reveals that gene expression exhibits distinct contributions to survival variation across cancers.

## Introduction

Cancer is one of the primary causes of death worldwide, leading to a growing severe threat to public health (Kyu et al., 2018; Roth et al., 2018; Siegel et al., 2019). Developing accurate models for prognostic prediction has long been an active research topic in cancer epidemiological study. Traditional prognostic prediction models consider only clinical and lifestyle information (e.g., tumor features, patient conditions, a few social and environmental factors) (Mallett et al., 2010); the predictive power is, hence, limited. The past few years have witnessed a great advent of high-throughput biotechnologies, and cancer research has already entered the era of precision medicine, in which patients’ individual genomic information is profiled to develop better diagnoses and treatment strategies that are tailored to their own tumors (Ashley, 2015; Collins and Varmus, 2015). As one of the largest genomics programs in the field of cancer biology, the Cancer Genome Atlas (TCGA) has generated multiple high-dimensional omic profiles (e.g., genomic, epigenomic, and transcriptomic biomarkers) on more than 11,000 patients with various types of cancer, offering an unprecedented opportunity for designing individualized treatments in terms of omics information (Weinstein et al., 2013; Tomczak et al., 2015).

Meanwhile, numerous prediction models were established by efficiently integrating available omics data sets for evaluating the progress and prognosis of tumors (Zhu et al., 2017), including head and neck squamous cell carcinoma (Shen et al., 2017a), oral squamous cell carcinoma (Shen et al., 2017b), and gastric cancer (Zhou et al., 2017). However, in terms of our literature review, we find many previous predictions only incorporate a small set of biomarkers into models in addition to clinical covariates (Supplementary Table S1); for example, only seven CpG-based methylation signatures were employed in Shen et al. (2017a), and alternatively, only some important biomarker information was extracted with dimensional reduction methods (e.g., principal component analysis, partial least squares, or variable selection methods; Zhao et al., 2014; Tang et al., 2017a, b) and was employed for prediction. This may be due to statistical difficulties when modeling high-dimensional omic data sets or due to the consideration of clinical application, in which a small set of biomarker predictors allow developing an implementable assay in clinical practice. From a methodological perspective, those existing approaches can be deemed to be sparse models as they explicitly assume only a small fraction of omics information is useful for prognostic prediction.

Although it has been shown that sparse models have the ability to improve the predictive accuracy of patients’ prognoses, we still have some concerns with regards to their prediction power due to the following limitations. First, the sparsity is a relatively strong assumption with little biological support. In practice, the truly genomic architecture for tumor progress and prognosis is rarely known in advance and is likely to vary dramatically among cancers (Zeng and Zhou, 2017; Zhu et al., 2017). Second, the performance of those prognostic methods generally depends on the correlation of selected biomarkers (e.g., CpG sites or mRNAs) with the target cancer. However, the selection of associated genes with cancer prognosis remains challenging (Chen and Hunter, 2005; Bouvard et al., 2009; El Ghissassi et al., 2009; Secretan et al., 2009; Plummer et al., 2016); as a consequence, the biomarkers incorporated into models are not necessarily highly predictive of tumor prognosis. Third, many potential biomarkers, which are excluded due to moderate or weak effect sizes, are possibly jointly important for survival variation (Eskin, 2015) and are actually useful for prognostic prediction (Yang et al., 2010; Golan and Rosset, 2011; Zhou et al., 2013). Therefore, the sparse prognostic prediction methods may be suboptimal.

Indeed, the prediction accuracy for prognostic prediction was recently shown to increase from 0.58, 0.62 to 0.64 when integrating 100, 300, or 5000 mRNAs into the model (Zhu et al., 2017), implying that, to some extent, incorporating more informative biomarkers can improve prediction performance. In contrast to sparse models, mixed-effects models, which include all available biomarkers, have shown promising accuracy in genetic prediction (Makowsky et al., 2011; Zhou et al., 2013; Zeng and Zhou, 2017). Therefore, a natural consideration is whether integrating a large number of biomarkers (e.g., genome-wide transcriptomic expressions) into prognostic models can further improve prediction performance. The difficulty is to determine how many genes should be included. Note that the inclusion of whole genome-wide transcriptomic expressions is one of the most commonly representative choices. It is also of great interest to empirically evaluate and compare the prediction performance of mixed models with sparse models when a large amount of omics information is available.

As it has been demonstrated that gene expressions possess the best predictive power for cancer prognostic assessment compared with other genomic measurements related to survival risk (Zhao et al., 2005, 2014; Zhu et al., 2017; Kim et al., 2018), in the present study, we only focus on this kind of omic information to explore how transcriptome data can be leveraged to improve prediction accuracy relative to prior sparse methods. First, a general Cox model with only clinical covariates is considered to be a benchmark. Next, we employ the sparse Cox model with an elastic net penalty and the linear mixed-effects Cox model to integrate clinical information as well as gene-expression levels. Moreover, we quantify the relative contribution of clinical and transcriptomic information to survival variance within the framework of a mixed-effects Cox model. In order to deeply evaluate the four kinds of Cox prediction models for the survival prognosis of cancer patients, we empirically apply them to 32 types of cancer in TCGA. Our results illustrate that the aggregation of genome-wide transcriptomic information can improve the prediction power substantially and further reveal that expression measurements show varying contributions to survival variation across cancers.

## Materials and Methods

### Overview of Four Prognostic Cox Prediction Models

We here offer an overview of the four kinds of Cox prognostic prediction models used in the present study with detailed descriptions of those models relegated to Supplementary File. Let ***X****_i_* be a *p*-dimensional vector for available clinical covariates (e.g., disease stage, age, and gender) for individual *i* in the TCGA data set (Hoadley et al., 2018) and assume each ***X*** is standardized to have mean zero and variance one. Denote the observed survival time by *t*_i_ and the true survival time by *T*_i_ with *d*_i_ indicating the censored status (i.e., *d_i_* = 1 if *T_i_* = *t*_i_, whereas *d_i_* = 0 if *T*_i_ < *t*_i_). We then employ the most widely used Cox model (Cox, 1972) $h\left(t_{\text{i}}|X_{\text{i}}\right)=h_{0}\left(t_{\text{i}}\right)e^{X_{\text{i}}^{\text{T}}a}$ to link survival risk with clinical information, where *h*_0_(*t*) is an arbitrary baseline hazard function and ***a*** = (*a*_1_, *a*_2,_…, *a*_p_) is a *p*-dimensional vector of effect sizes for covariates.

Let *G*_i_ be an *m*-dimensional vector for a set of expression levels for individual *i* and assume each expression is standardized. The Cox model including both *X*_i_ and *G*_i_ is written as $h\left(t_{\text{i}}|X_{\text{i}},G_{\text{i}}\right)=h_{0}\left(t_{\text{i}}\right)e^{X_{\text{i}}^{\text{T}}a+G_{\text{i}}^{\text{T}}b}$ with *b* = (*b*_1_, *b*_2,_ …, *b*_m_) an *m*-dimensional vector of effect sizes for expressions. Because of the high dimension (i.e., *m* ≫ *n*, see Table 1), we have to apply regularization methods for parameter estimation to avoid model overfitting (Fan and Li, 2001; Zou and Hastie, 2005; Tibshirani, 2011; Hastie et al., 2015) and only focus on the Cox model with the lasso or elastic net penalty (denoted by coxlasso or coxenet, respectively) (Zou and Hastie, 2005; Zeng et al., 2017). We adopt the coordinate descent algorithm (Friedman et al., 2010) to fit coxenet and select the optimal tuning parameter via a subsampling strategy (Hastie et al., 2009). Note that, with a suitable tuning parameter, most of the effect sizes for gene expressions in coxlasso or coxenet would be shrunk to be exactly zero, leading to the so-called sparse model. We set *α* = 0.50 in the elastic net penalty as done in prior work (Gamazon et al., 2015; Zeng et al., 2017) and implement model fitting with the R glmnet (version 2.0-5) package (Friedman et al., 2010).

**TABLE 1:** Basic information of the raw data sets and quality control for 32 cancers in TCGA used in the present study.

Cancer	Initial data			After combined (*n*)	After quality control (*n* and *m*)	
	Gene expression (*n* and *m*)		Clinical (*n*)			
Adrenocortical Carcinoma (ACC)	79	20,530	92	79	77	19,194
Bladder Urothelial Carcinoma (BLCA)	426	20,530	436	425	400	20,164
Breast Invasive Carcinoma (BRCA)	1,218	20,530	1247	1215	901	20,131
Cervical Squamous Cell Carcinoma and Endocervical Adenocarcinoma (CESC)	308	20,530	313	307	287	19,556
Cholangiocarcinoma (CHOL)	45	20,530	45	45	36	19,352
Colon Adenocarcinoma (COAD)	329	20,530	551	328	270	18,707
Lymphoid Neoplasm Diffuse Large B-cell Lymphoma (DLBC)	48	20,530	48	48	41	18,125
Esophageal Carcinoma (ESCA)	196	20,530	204	196	159	19,501
Glioblastoma Multiforme (GBM)	172	20,530	629	172	143	17,996
Head and Neck Squamous Cell Carcinoma (HNSC)	566	20,530	604	566	440	19,526
Kidney Chromophobe (KICH)	91	20,530	91	91	65	18,104
Kidney Renal Clear Cell Carcinoma (KIRC)	606	20,530	945	606	526	19,212
Kidney Renal Papillary Cell Carcinoma (KIRP)	323	20,530	352	323	256	19,309
Acute Myeloid Leukemia (LAML)	173	20,530	200	173	161	16,718
Brain Lower Grade Glioma (LGG)	530	20,530	530	530	502	17,308
Liver Hepatocellular Carcinoma (LIHC)	423	20,530	438	422	343	19,382
Lung Adenocarcinoma (LUAD)	576	20,530	706	576	486	20,068
Lung Squamous Cell Carcinoma (LUSC)	553	20,530	626	552	481	20,004
Mesothelioma (MESO)	87	20,530	87	87	81	19,463
Ovarian Serous Cystadenocarcinoma (OV)	308	20,530	630	308	290	19,404
Pancreatic Adenocarcinoma (PAAD)	183	20,530	196	183	175	19,609
Pheochromocytoma and Paraganglioma (PCPG)	187	20,530	187	187	176	17,886
Prostate Adenocarcinoma (PRAD)	550	20,530	566	550	483	18,456
Rectum Adenocarcinoma (READ)	105	20,530	186	105	81	18,316
Sarcoma (SARC)	265	20,530	271	264	258	20,083
Skin Cutaneous Melanoma (SKCM)	474	20,530	477	481	409	19,638
Stomach Adenocarcinoma (STAD)	450	20,530	580	450	379	19,797
Thyroid Carcinoma (THCA)	572	20,530	580	570	497	18,027
Thymoma (THYM)	122	20,530	126	122	117	18,470
Uterine Corpus Endometrial Carcinoma (UCEC)	201	20,530	596	201	172	19,918
Uterine Carcinosarcoma (UCS)	57	20,530	57	50	50	19,059
Uveal Melanoma (UVM)	80	20,530	80	80	76	17,239

*n represents the sample size, m is the number of gene expressions used in our final analysis.*

Unlike coxenet, which assumes only a few of genes are involved in the survival risk, the linear mixed-effects Cox model (denoted by coxlmm) explicitly supposes that all genes may be implicated in cancer progress and have nonzero effects (Zhou et al., 2013; Ott, 2016) (i.e., $b_{\text{j}}∼N\left(0,\sigma_{\text{b}}^{2}\right)$ with $\sigma_{\text{b}}^{2}$ the variance). We fit coxlmm with the R coxme (version 2.2-10) package (Therneau, 2019) via the Laplace approximation method based on the second order Taylor series expansion (Therneau et al., 2003).

### Relative Overall Importance of Clinical and Transcriptomic Information

To quantify the relative overall importance/contribution of clinical and transcriptomic information to survival phenotypes (Korsgaard et al., 1998; Yazdi et al., 2002; Gorfine et al., 2017), we first make a log-transformation for the hazard function and then define two quantities: the proportion of the survival variation explained by the clinical information (PCE) and the proportion of the survival variation explained by the transcriptome information (PGE). The summation of PCE and PGE is the proportion of the survival variation explained (PVE) by currently available clinical and transcriptomic information together. We apply the Jackknife method to yield the confidence interval for PCE or PGE (Efron and Tibshirani, 1994). Further computational details for PCE and PGE are described in Supplementary File.

### TCGA Cancer Data Sets and Quality Control

We now apply these Cox models to cancer data sets publicly available from TCGA (Hoadley et al., 2018). For those cancers, we obtained their clinical information and RNAseq expression levels. Note that, for comparison across all types of cancer in TCGA, we employed pan-cancer normalized gene expression RNAseq (IlluminaHiSeq) data sets provided by UCSC Xena^1^. We selected overall survival time and status and included age, gender, and pathologic tumor stage because only these clinical variables are available for most of the patients. When the pathologic tumor stage is unavailable, we alternatively employed the clinical stage (i.e., CESC and OV) or histological grade (i.e., LGG). All three stage variables are missing for five cancer data sets (e.g., SARC). Cancer-specific covariates were also considered for some cancers; for example, two binary variables (i.e., the status of estrogen or progesterone receptor) were added to BRCA.

For each cancer, we first merged clinical information and gene expressions measured from the primary cancer tissue and then excluded samples soaked in the formalin-fixed paraffin-embedded tissue. Cancers with a sample size greater than 175 and a proportion of censored event less than 15% were defined as low-censored data sets. To some extent, these two threshold values (i.e., the sample size of 175 and the proportion of 15%) were selected arbitrarily. Testicular germ cell tumor (TGCT) was excluded as nearly all TGCT patients were alive during follow-up. Therefore, among the 33 publicly available types of TCGA cancer, we reserved 32 cancer data sets in the subsequent prediction analysis and divided them into 16 low-censored and 16 high-censored cancer data sets according to our criterion above. In addition, we removed genes with more than 50% zero expressions and variances smaller than 20% quantile of expressions (Tang et al., 2017a; Yu et al., 2019). Finally, we standardized the remaining gene-expression levels and clinical covariates. The data sets used in this study are summarized in Table 2.

**TABLE 2:** Summary information of 32 types of cancer in TCGA.

Cancer	Age	Female/Male	Median survival time			Stage or grade (1/2/3/4/5)
			All	Event	Censor	
ACC	46.6 ± 15.8	48/29	38.5	18.5	45.7	9/37/16/15
BLCA	68.1 ± 10.6	105/295	17.6	13.6	20.9	2/129/137/132
BRCA	58.5 ± 13.2	1060/0	27.2	37.9	25.0	178/605/245/19/13
CESC	48.0 ± 13.6	287/287	21.1	20.8	22.8	157/67/43/20
CHOL	63.0 ± 12.9	20/16	21.2	16.4	31.0	19/9/1/7
COAD	65.2 ± 13.3	121/149	21.6	14.5	22.0	45/109/78/38
DLBC	55.1 ± 14.7	22/19	31.1	19.6	31.8	8/17/5/11
ESCA	62.4 ± 11.9	24/135	13.2	12.9	13.4	18/78/55/8
GBM	59.7 ± 13.5	48/95	11.3	12.6	7.9	NA
HNSC	60.9 ± 12.1	120/320	21.4	14.2	27.4	26/70/81/263
KICH	51.2 ± 14.1	27/38	73.9	28.1	89.2	20/25/14/6
KIRC	60.7 ± 12.1	186/340	39.7	27.0	48.2	265/56/122/83
KIRP	61.6 ± 12.0	67/189	24.1	20.5	24.9	170/20/51/15
LAML	55.8 ± 16.3	74/87	11.0	9.0	23.0	NA
LGG	43.0 ± 13.4	224/278	21.6	26.8	20.5	0/241/261/0
LIHC	58.7 ± 13.5	109/234	18.7	12.5	20.9	171/85/82/5
LUAD	65.4 ± 10.0	263/223	21.4	20.1	21.6	263/117/80/26
LUSC	67.2 ± 8.5	126/355	21.3	17.8	24.1	237/154/83/7
MESO	62.9 ± 9.9	16/65	16.4	15.0	38.4	9/15/42/15
OV	59.3 ± 11.0	290/0	31.3	35.0	24.7	1/18/233/38
PAAD	64.6 ± 11.0	79/96	15.2	12.9	16.7	21/147/3/4
PCPG	47.3 ± 15.1	99/77	25.1	14.9	25.4	NA
PRAD	61.0 ± 6.8	0/483	30.3	43.7	30.1	NA
READ	63.2 ± 12.1	37/44	24.6	19.7	25.1	10/26/33/12
SARC	60.7 ± 14.6	140/118	31.3	22.0	35.9	NA
SKCM	58.8 ± 15.6	154/255	32.9	31.6	34.1	77/139/170/23
STAD	65.3 ± 10.6	136/243	14.5	11.5	18.6	53/121/166/39
THCA	47.2 ± 15.8	363/134	31.0	33.6	31.0	282/52/110/53
THYM	58.1 ± 13.1	56/61	40.1	28.0	40.7	35/61/15/6
UCEC	65.6 ± 11.4	172/0	22.1	20.5	22.3	93/24/45/10
UCS	69.7 ± 9.4	50/0	20.0	16.5	26.9	21/4/17/8
UVM	62.8 ± 13.0	35/41	25.0	19.4	26.6	0/37/35/4

### Model Comparison and Implementation

Following prior work (Zeng and Zhou, 2017), we conducted a subsampling strategy to evaluate prediction performance. We performed 100 Monte Carlo cross-validations (MCCVs) by randomly dividing the total cancer data set into two parts with 80% of the samples as training data and the remaining 20% as test data. Then, we fitted prediction models in the training data and calculated Harrell’s concordance index (C-index) to measure the prediction accuracy in the test data (Harrell et al., 1982). The C-index is a measure of goodness of fit for survival models, which produce risk scores. Specifically, assume all the subjects under consideration are randomly paired. In survival analysis, if one of them has a longer survival time and predicted survival time or predicted survival probability than the other, it is said that the predicted result is consistent with the actual result, which is referred to as the concordance. Then, Harrell’s C-index can be simply calculated as the proportion of the number of concordant pairs in the total number of concordant and discordant pairs. A C-index statistic of 0.5 means complete inconsistency, indicating the model has little predictive ability, and a C-index statistic of one represents complete consistency, indicating the prediction results of the model are completely in line with the actual situations. To yield the prediction gain of one model (say M1) relative to another model (say M2), we computed (*C*_M2_-*C*_M1_)/*C*_M1_ with *C*_M2_ and *C*_M1_ the C-index statistic values for the two models.

In our prediction analysis, as is shown, compared with other models (e.g., coxenet), coxlmm displays a robust predictive performance by integrating whole gene-expression profiles. However, it remains unknown whether the improved performance of coxlmm is due to the incorporation of useful transcriptomic information or just a consequence of overfitting owing to more parameters being involved. Therefore, in order to further validate the prediction performance of coxlmm, for each cancer, we simultaneously generated a new data set by only permuting expression levels. Doing this is equivalent to adding a set of *noise* expressions into the prediction model. For each type of cancer, we re-performed coxlmm in terms of MCVC based on unchanged covariates (and survival time and status) and permuted expressions. Theoretically, the newly permuted expression levels are little predictive, and the resulting C-index statistic is similar to or even lower than that obtained with the total original data sets because the permuted expressions are redundant for prediction once they are included into models. In order to further validate our assumption that a large number of weak-effect genes also may be useful in the prediction of cancer prognoses and to examine whether the whole transcriptome model is optimal, we compare the accuracy of models with different sets of genes via coxlmm based on 20 replications of MCCV. We considered two schemes to select genes: first, genes were randomly selected; second, genes were selected in terms of marginal *p*-values of the univariate Cox model in the training data. Finally, the prediction accuracy was evaluated in the testing data set via C-index.

## Results

### Evaluation of Prognostic Prediction

A total of 32 TCGA cancers are included in our analysis, and the summary results of those cancers are displayed in Tables 1, 2. Here, we mainly consider three covariates (i.e., age, sex, and stage or grade) if they are available for some cancers and make a comparison for the four Cox models in terms of the difference in C-index statistic. We observe that the clinical information displays varying prognostic prediction ability across cancers. Specifically, the C-index statistic ranges from 0.54 for PAAD to 0.81 for KIRP with an average of 0.66 for the low-censored cancers; the C-index statistic ranges from 0.46 for PRAD to 0.81 for KIRP with an average of 0.66 for all 32 cancers.

### Prediction Performance for the 16 Low-Censored TCGA Cancers

**FIGURE 1:**
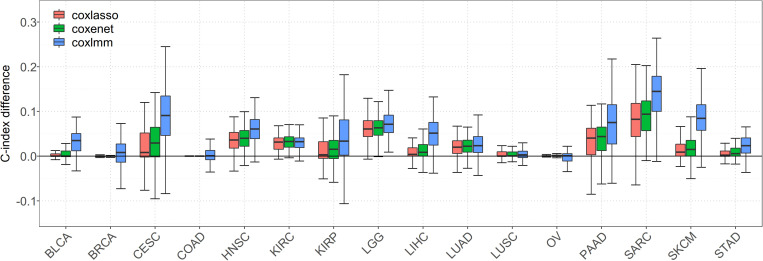
Comparison of predictive performance of four models in 16 low-censored cancers. Performance is measured by C-index difference with respect to Cox model with only clinical covariates; therefore, a negative value (i.e., values below the horizontal line) indicates worse performance than the Cox model with only clinical covariates, and the predictive performance was assessed across 100 replicates.

We first focus on the performance of 16 special cancers that have a relatively low-censored rate and, thus, are of our main interest in prognostic prediction. It is worth noting that, when gene expressions are incorporated into the model, the prediction performance is substantially improved for almost all the cancers (Figure 1). Compared with the Cox model with clinical information alone, the averages of the prediction gain for coxlasso, coxenet, and coxlmm are 2.4, 4.2, and 7.2% across cancers, respectively (Supplementary Table S2).

In particular, a much more significant elevation of C-index statistic is discovered for several cancers (e.g., SARC, SKCM, LGG, PAAD, and HNSC) for coxlasso, coxenet, and coxlmm. For example, compared to the Cox model with only clinical covariates, coxlasso displays the maximum improvement for SARC (13.8%), followed by LGG (7.8%), PAAD (6.4%), and HNSC (6.0%). The corresponding gains are 15.6, 7.6, 7.4, and 6.8% for coxenet and 22.8, 8.9, 14.8, and 10.2% for coxlmm for these four cancers, respectively. Furthermore, the prediction improvement for coxlmm is also evident for other cancers, including CESC (14.3%), LIHC (7.9%), KIRP (6.2%), and BLCA (6.2%). Nevertheless, relative to the clinical information–only Cox model, we find there is nearly no remarkable increase in the prediction accuracy of coxlmm, coxenet, or coxlasso for COAD and OV after incorporating transcriptomic information.

Finally, we find coxlmm always has a comparable or the best prediction performance among the methods. For example, coxlmm demonstrates an average of 2.8% prediction improvement across all 16 cancers compared with coxenet with the maximum gain for CESC (9.1%, from 4.8% for coxenet to 14.3% for coxlmm relative to the clinical information–only Cox model), followed by PAAD (6.9%, from 7.4% for coxenet to 14.8% for coxlmm relative to the clinical information–only Cox model). In addition, coxlmm also displays an obvious gain in the prediction performance for LIHC and SARC relative to coxenet with 6.3 and 6.1% increases in the C-index statistic, respectively. These findings imply that the Cox model incorporating all the transcriptome information can further increase prediction ability relative to that including only a small set of important genes.

### Prediction Performance for All 32 TCGA Cancers

With regards to all 32 cancers, compared with the Cox model with clinical information alone, the maximum gain is observed for MESO (35.6% for coxlasso, 39.6% for coxenet, and 50.0% for coxlmm) (Supplementary Figures S1, S2). The averages of prediction gain for coxlasso, coxenet, and coxlmm are 2.7, 3.8, and 5.8% across cancers, respectively, and are lower than performance for those low-censored cancer data sets. Compared to coxlasso and coxenet, the average gains of coxlmm are 3.0 and 1.3%, which are also lower than performance for those low-censored cancer data sets.

### Prediction Performance of Coxlmm With Permuted Data Sets

**FIGURE 2:**
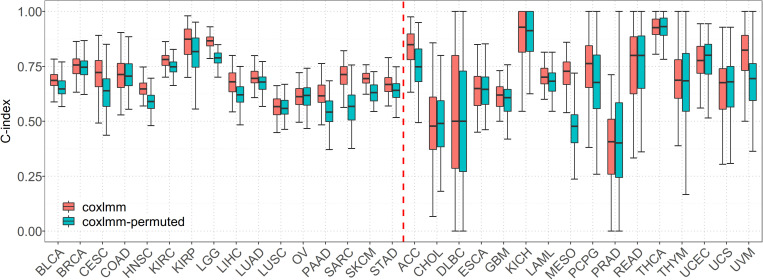
Performance comparison of coxlmm using the original data sets (denoted by coxlmm) and the permuted data sets (denoted by coxlmm-permuted) across the 32 TCGA cancers. The cancers located to the left of the red dotted line are low-censored, and the cancers on the right side are high-censored.

When predicting with the permuted data set, we find that, compared with coxlmm with permuted expressions, the original coxlmm model has an average of 4.7% higher C-index statistics across the 16 low-censored cancers and shows an average of 9.0% performance gain for the five most promising cancers with the highest PVE (i.e., CESC, LGG, PAAD, SARC, and SKCM). As anticipated, coxlmm even demonstrates slightly worse performance using those permuted cancer data sets compared with the general Cox model with only covariates. The detailed performance for each permuted cancer is showed in Figure 2. These observations suggest that integrating all the gene expressions into coxlmm does improve the prediction performance and the predictive gain of coxlmm relative to other existing models is not by chance alone.

### Estimates of PCE and PGE

**FIGURE 3:**
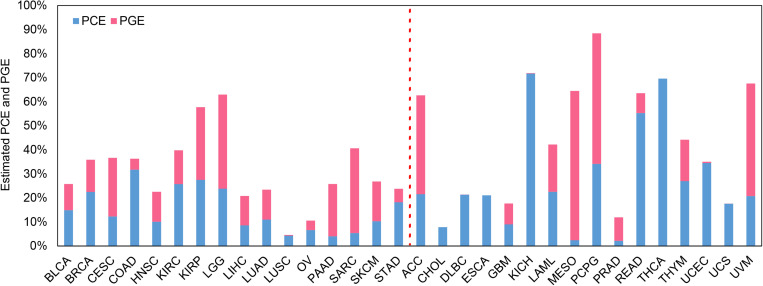
Estimated PCE and PGE for the 32 TCGA cancer types. The cancers located to the left of the red dotted line are low-censored, and the cancers on the right side are high-censored. PCE represents the proportion of the survival variation explained by the clinical information alone. PGE represents the proportion of the survival variation explained by the transcriptome information alone.

The estimated PCE and PGE are shown in Figure 3 with the confidence intervals displayed in Supplementary Figure S3 and Supplementary Table S3. Again, here we mainly discuss the estimates for the 16 low-censored cancers. Several interesting findings can be observed. First, except for LUSC (PVE = 4.6%), it is shown that both the clinical and transcriptomic information plays an important role in the survival variation of TCGA cancers (e.g., PVE is greater than 10.0%) with PVE ranging from 10.6% for OV to 63.0% for LGG. Second, for five cancers (i.e., LGG, CESC, PAAD, SKCM, and SARC), PGE is 15.3, 12.1, 17.8, 6.3, and 29.9% higher than PCE (with an average of 16.3%), suggesting the transcriptomic information is relatively more important than the clinical information for those cancers, and the prediction performance would improve substantially if gene-expression levels are included (see results above and Supplementary Table S1; the average increase in C-index is 3.8% for coxlasso, 8.7% for coxenet, and 14.1% for coxlmm for the five cancers). On the other hand, PCE is higher than PGE for four cancers (i.e., BRCA, COAD, KIRC, and STAD), implying that the prediction performance would improve little when integrating transcriptomic information (see results above and Supplementary Table S1; the average increase in C-index is 1.3% for coxlasso, 1.1% for coxenet, and 1.7% for coxlmm for the four cancers). Finally, the remaining cancers (i.e., BLCA, HNSC, KIRP, LIHC, LUAD, LUSC, and OV) show similar estimates for PCE and PGE. We also perform coxlmm to estimate the PCE and PGE in the other 16 high-censored cancers. However, their results are rather unstable, reflected by the wide confidence intervals (Supplementary Figure S3).

### Influence of Different Numbers of Genes Included in Coxlmm

**FIGURE 4:**
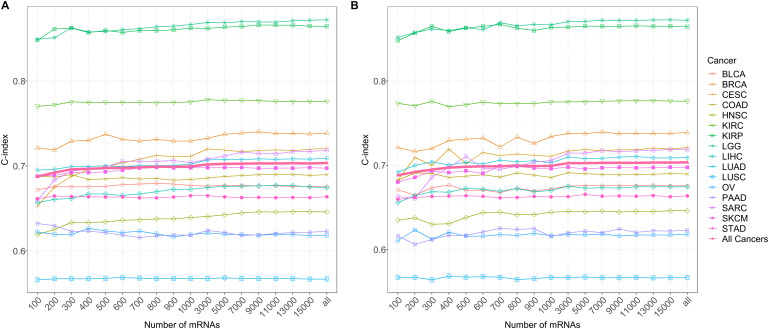
**(A)** The predictive accuracy with a different number of genes after sorting by importance for 16 low-censored cancers. **(B)** The predictive accuracy with a different number of randomly selected genes for 16 low-censored cancers. The thick pink line represents the average C-index of all cancers.

It is found that the prediction performance of coxlmm with different numbers of genes is cancer-specific (Figure 4 and Supplementary Figure S4). For example, integrating more genes in several cancers (e.g., LUSC, LGG, and KIRC) does not lead to the improvement of prediction although doing this indeed increases the prediction performance for other cancers (e.g., CESC, HNSC, and SARC). Generally, no matter how the genes were selected, the prediction accuracy of the model (denoted by thick lines in Figure 4) shows a slightly increasing trend with the included genes and approaches the peak when 3000 genes were employed.

## Discussion and Conclusion

In this study, we focused on four Cox prediction approaches (i.e., the general Cox model, coxlasso, coxenet, coxlmm) and systematically assessed the prognostic values of transcriptomes with the publicly available TCGA pan-cancer data sets (Hoadley et al., 2018). It should be first emphasized that prediction performance is cancer-specific; that is, the prediction accuracy, quantified by C-index statistic, is substantially different across cancer types, consistent with the conclusions found in prior studies (Zhao et al., 2014; Zhu et al., 2017). In particular, our results indicate that coxlmm holds preferable predictive performance compared with the other three methods and reveal that integrating both clinical and transcriptomic information can elevate the prediction accuracy greatly. Furthermore, it also implies that incorporating a large number of gene expressions rather than a small set of selective genes can obtain more gain in prediction accuracy, which generally continues to improve with the increasing number of genes included. We further assess the contributions of clinical covariates and gene expressions to the survival variation within the framework of coxlmm. We can expect that PCE and PGE together should capture almost all the survival variation explained by available information as it has been illustrated that no significant power gain can be achieved by combining other omic measurements into clinical covariates except for gene expression (Zhao et al., 2014). This result is biologically informative and illustrates that the performance of prediction models would improve considerably when transcriptomic information accounts for a large proportion of the variation as quantified by PGE.

Note that our main objective here is not to explore how to efficiently integrate multiple omic profiles into prediction models (Zhao et al., 2014; Yang et al., 2016, 2018; Huang et al., 2017; Zhu et al., 2017) although this is a very interesting and important research area. Instead, we aim to evaluate how a single omic profile (i.e., gene expression) can be utilized to achieve better prognostic prediction in cancers. To the best of our knowledge, the present study is among the first work to comprehensively investigate prognostic prediction with the transcriptome profile on dozens of cancer types (i.e., a total of 32 common complex cancers). Therefore, our results provide complementary insights for prior work in which gene expressions were already shown to be very predictive for cancer prognosis assessment relative to other omic profiles (Zhao et al., 2014; Zhu et al., 2017). In addition, the analysis we carried out here also can be applicable to other types of omic measurements.

We finally highlight some interesting topics for future exploration. First, as mentioned before, we only consider transcriptomic information in our analysis and ignore other omics data sets (e.g., copy number alteration and DNA methylation), and we also cannot incorporate more useful clinical covariates (e.g., smoking or drinking history) as that information is missing during the period of data collection in TCGA. Thus, the performance of our prediction models may be underestimated. Integrating multi-omics information into the model can unquestionably achieve better prediction accuracy and has the potential to provide a deeper understanding of the mechanism of tumor progression.

Second, although TCGA includes many cancer types, its effective sample size is still relatively small, and the censored proportion is high (Hoadley et al., 2018), which may inevitably attenuate the prediction accuracy and undermine our ability to construct prognostic models that can be applicable in practice. In addition, the small sample size may also lead to unstable estimation for PCE and PGE. Thus, the external validation of our models with larger a sample size is warranted.

Third, in the present study, the proposed coxlmm actually can be treated to be a kernel machine learning–based prediction method, and only the linear kernel was considered here. The selection of optimal kernel function to measure the similarity among transcriptomes and to better improve prognostic evaluation needs further investigation (Yang et al., 2016, 2018). In addition, coxlmm also can be viewed as a penalty-type Cox model with ridge regularization (denoted by coxridge), which has the square penalty on effect sizes. We compared coxlmm and coxridge in 16 low-censored cancers and demonstrated that two methods generally had consistent performance (Supplementary Figure S5) although sometimes coxlmm performed slightly better in several cancers.

Fourth, compared with coxlasso and coxenet, we indeed observed that coxlmm, integrating more genes, had a better prediction performance. In general, the predictive accuracy of coxlmm would continue to improve when more genes were integrated. However, the model with whole transcriptomic information may be suboptimal for some cancers (e.g., CESC, HNSC, and SARC) because of the inclusion of redundant genes that were not useful for prediction. Therefore, exploring adaptive models that can select optimal genes is an interesting direction in the future. In addition, following the idea of functional gene enrichment analysis, we can first classify genes into various groups in terms of similar function in the same pathway and then conduct a separate prediction in each group and finally aggregate the individual predictions into an omnibus prediction with some reasonable weighted manners. This will probably be a considerably promising avenue in our further investigation.

Fifth, we find that the mixed-effects Cox model (i.e., coxlmm) sometimes showed low prediction accuracy for some cancers (e.g., KIRC) compared with the sparse Cox model (i.e., coxlasso and coxenet) although it demonstrated promising performance for most of the TCGA cancer types, indicating the two kinds of models have their own advantages depending on the omic architecture of a specific cancer type (Zhou et al., 2013; Tang et al., 2017a, b; Zeng and Zhou, 2017). A natural extension is to combine the two models and generate a hybrid of the mixed and sparse prediction approaches by following prior work in genetic prediction (Zhou et al., 2013; Zeng and Zhou, 2017). The mixed-sparse Cox model is anticipated to provide better prognostic prediction for different cancer types. Simultaneously, this type of model has the ability to select significant genes that may be biologically important for cancers.

Overall, the present study demonstrates that the aggregation of genome-wide transcriptomic information can lead to great improvement in prediction accuracy, but the prediction performance is cancer-specific and varies across cancer types. It further reveals that gene expression shows varying contributions to survival variation across cancers.

## Data Availability Statement

The TCGA data sets can be publicly available from https://xenabrowser.net/. Additionally, all data generated or analyzed during this study are included in this article/Supplementary Material. The codes that were employed to perform coxlasso, coxenet and coxlmm can be available from https://github.com/biostatYu/coxlmm.

## Author Contributions

PZ and SH conceived the idea for the study. PZ, XY, and TW obtained the data, performed the data analyses, and interpreted the results of the data analyses. PZ and XY drafted the manuscript, and all authors approved the manuscript and provided relevant suggestions.

## Conflict of Interest

The authors declare that the research was conducted in the absence of any commercial or financial relationships that could be construed as a potential conflict of interest.

## Footnotes

1 https://xenabrowser.net/

## Abbreviations

Coxenet: the Cox model with elastic net penalty
coxlasso: Cox model with lasso penalty
coxlmm: the mixed Cox model
MCCV: Monte Carlo Cross Validation; the abbreviations of cancers were summarized in Table 1
PCE: the proportion of the survival variation explained by the clinical information alone
PGE: the proportion of the survival variation explained by the transcriptome information alone
PVE: proportion of the survival variation explained
TCGA: the Cancer Genome Atlas.

## References

[B1] Ashley E. A. (2015). The precision medicine initiative: a new national effort. *JAMA* 313 2119–2120. 25928209 10.1001/jama.2015.3595

[B2] Bouvard V. Baan R. Straif K. Grosse Y. Secretan B. El Ghissassi F. (2009). A review of human carcinogens–Part B: biological agents. *Lancet. Oncol.* 10 321–322. 10.1016/s1470-2045(09)70096-8 19350698

[B3] Chen Y. C. Hunter D. J. (2005). Molecular epidemiology of cancer. *CA Cancer J. Clin.* 55 45–54. 15661686 10.3322/canjclin.55.1.45

[B4] Collins F. S. Varmus H. (2015). A new initiative on precision medicine. *New England J. Med.* 372 793–795. 10.1056/nejmp1500523 25635347 PMC5101938

[B5] Cox D. R. (1972). Regression models and Life-tables. *J. Royal Stat. Soc. Ser. B (Methodological)* 34 187–220.

[B6] Efron B. Tibshirani R. J. (1994). *An Introduction to the Bootstrap.* Boca Raton, FLA: CRC press.

[B7] El Ghissassi F. Baan R. Straif K. Grosse Y. Secretan B. Bouvard V. (2009). A review of human carcinogens—part D: radiation. *Lancet. Oncol.* 10 751–752. 10.1016/s1470-2045(09)70213-x 19655431

[B8] Eskin E. (2015). Discovering genes involved in disease and the mystery of missing heritability. *Commun. ACM* 58 80–87. 10.1145/2817827

[B9] Fan J. Li R. (2001). Variable selection via nonconcave penalized likelihood and its oracle properties. *J. Am. Stat. Assoc.* 96 1348–1360. 10.1198/016214501753382273 12611515

[B10] Friedman J. Hastie T. Tibshirani R. (2010). Regularization paths for generalized linear models via coordinate descent. *J. Stat. Softw.* 33 1–22. 20808728 PMC2929880

[B11] Gamazon E. R. Wheeler H. E. Shah K. P. Mozaffari S. V. Aquino-Michaels K. Carroll R. J. (2015). A gene-based association method for mapping traits using reference transcriptome data. *Nat. Genet.* 47 1091–1098. 10.1038/ng.3367 26258848 PMC4552594

[B12] Golan D. Rosset S. (2011). Accurate estimation of heritability in genome wide studies using random effects models. *Bioinformatics* 27 i317–i323. 10.1093/bioinformatics/btr219 21685087 PMC3117387

[B13] Gorfine M. Berndt S. I. Chang-Claude J. Hoffmeister M. Le Marchand L. Potter J. (2017). Heritability estimation using a regularized regression approach (HERRA): applicable to continuous, dichotomous or age-at-onset outcome. *PLoS One* 12:e0181269. 10.1371/journal.pone.0181269 28813438 PMC5559077

[B14] Harrell F. E. Califf R. M. Pryor D. B. Lee K. L. Rosati R. A. (1982). Evaluating the yield of medical tests. *JAMA* 247 2543–2546. 10.1001/jama.247.18.2543 7069920

[B15] Hastie T. Tibshirani R. Friedman J. (2009). *The Elements of Statistical Learning: Data mining, Inference, and Prediction.* Berlin: Springer Science & Business Media.

[B16] Hastie T. Tibshirani R. Wainwright M. (2015). *Statistical Learning with Sparsity: the lasso and Generalizations.* New York, NY: CRC Press.

[B17] Hoadley K. A. Yau C. Hinoue T. Wolf D. M. Lazar A. J. Drill E. (2018). Cell-of-origin patterns dominate the molecular classification of 10,000 tumors from 33 types of cancer. *Cell* 173 291–304.e6. 29625048 10.1016/j.cell.2018.03.022 PMC5957518

[B18] Huang S. Chaudhary K. Garmire L. X. (2017). More is better: recent progress in multi-omics data integration methods. *Front. Genet.* 8:84. 10.3389/fgene.2017.00084 28670325 PMC5472696

[B19] Kim Y. Kang Y. S. Seok J. (2018). GAIT: gene expression analysis for interval time. *Bioinformatics* 34 2305–2307. 10.1093/bioinformatics/bty111 29509896

[B20] Korsgaard I. R. Madsen P. Jensen J. (1998). Bayesian inference in the semiparametric log normal frailty model using Gibbs sampling. *Genet. Select. Evol.* 30 241–256.

[B21] Kyu H. H. Abate D. Abate K. H. Abay S. M. Abbafati C. Abbasi N. (2018). Global, regional, and national disability-adjusted life-years (DALYs) for 359 diseases and injuries and healthy life expectancy (HALE) for 195 countries and territories, 1990–2017: a systematic analysis for the global burden of disease study 2017. *Lancet* 392 1859–1922. 30415748 10.1016/S0140-6736(18)32335-3 PMC6252083

[B22] Makowsky R. Pajewski N. M. Klimentidis Y. C. Vazquez A. I. Duarte C. W. Allison D. B. (2011). Beyond missing heritability: prediction of complex traits. *PLoS Genet.* 7:e1002051. 10.1371/journal.pgen.1002051 21552331 PMC3084207

[B23] Mallett S. Royston P. Waters R. Dutton S. Altman D. G. (2010). Reporting performance of prognostic models in cancer: a review. *BMC Med.* 8:21. 10.1186/1741-7015-8-21 20353579 PMC2857810

[B24] Ott J. (2016). Polygenic models for risk prediction in human genetics. *Hum. Hered.* 80 162–164. 10.1159/000447593 27576755

[B25] Plummer M. De Martel C. Vignat J. Ferlay J. Bray F. Franceschi S. (2016). Global burden of cancers attributable to infections in 2012: a synthetic analysis. *Lancet Global Health* 4 e609–e616. 10.1016/s2214-109x(16)30143-7 27470177

[B26] Roth G. A. Abate D. Abate K. H. Abay S. M. Abbafati C. Abbasi N. (2018). Global, regional, and national age-sex-specific mortality for 282 causes of death in 195 countries and territories, 1980–2017: a systematic analysis for the global burden of disease study 2017. *Lancet* 392 1736–1788. 30496103 10.1016/S0140-6736(18)32203-7 PMC6227606

[B27] Secretan B. Straif K. Baan R. Grosse Y. El Ghissassi F. Bouvard V. (2009). A review of human carcinogens–Part E: tobacco, areca nut, alcohol, coal smoke, and salted fish. *Lancet. Oncol.* 10 1033–1034. 10.1016/s1470-2045(09)70326-2 19891056

[B28] Shen S. Bai J. Wei Y. Wang G. Li Q. Zhang R. (2017a). A seven-gene prognostic signature for rapid determination of head and neck squamous cell carcinoma survival. *Oncol. Rep.* 38 3403–3411. 29130107 10.3892/or.2017.6057 PMC5783586

[B29] Shen S. Wang G. Shi Q. Zhang R. Zhao Y. Wei Y. (2017b). Seven-CpG-based prognostic signature coupled with gene expression predicts survival of oral squamous cell carcinoma. *Clin. Epigenet.* 9:88. 10.1186/s13148-017-0392-9 PMC5571486 28852427

[B30] Siegel R. L. Miller K. D. Jemal A. (2019). Cancer statistics, 2019. *CA Cancer J. Clin.* 69 7–34. 30620402 10.3322/caac.21551

[B31] Tang Z. Shen Y. Li Y. Zhang X. Wen J. Qian C. A. (2017a). Group spike-and-slab lasso generalized linear models for disease prediction and associated genes detection by incorporating pathway information. *Bioinformatics* 34 901–910. 10.1093/bioinformatics/btx684 29077795 PMC5860634

[B32] Tang Z. Shen Y. Zhang X. Yi N. (2017b). The spike-and-slab lasso Cox model for survival prediction and associated genes detection. *Bioinformatics* 33 2799–2807. 10.1093/bioinformatics/btx300 28472220 PMC5870779

[B33] Therneau T. M. (2019). *coxme: Mixed Effects Cox Models. R package Version* 2.2-14. https://CRAN.R-project.org/package=coxme.

[B34] Therneau T. M. Grambsch P. M. Pankratz V. S. (2003). Penalized survival models and frailty. *J. Computat. Graph. Stat.* 12 156–175.

[B35] Tibshirani R. (2011). Regression shrinkage and selection via the lasso. *J. Royal Stat. Soc.* 73 267–288. 10.1111/j.2517-6161.1996.tb02080.x

[B36] Tomczak K. Czerwińska P. Wiznerowicz M. (2015). The Cancer Genome Atlas (TCGA): an immeasurable source of knowledge. *Contemp. Oncol.* 19 A68–A77. 10.5114/wo.2014.47136 PMC4322527 25691825

[B37] Weinstein J. N. Collisson E. A. Mills G. B. Shaw K. R. M. Ozenberger B. A. Ellrott K. (2013). The cancer genome atlas pan-cancer analysis project. *Nat. Genet.* 45 1113. 10.1038/ng.2764 24071849 PMC3919969

[B38] Yang H. Cao H. He T. Wang T. Cui Y. (2018). Multilevel heterogeneous omics data integration with kernel fusion. *Brief. Bioinform.* 21 156–170. 10.1093/bib/bby115 30496340

[B39] Yang H. Li S. Cao H. Zhang C. Cui Y. (2016). Predicting disease trait with genomic data: a composite kernel approach. *Brief. Bioinform.* 18 591–601. 10.1093/bib/bbw043 27255915

[B40] Yang J. Benyamin B. Mcevoy B. P. Gordon S. Henders A. K. Nyholt D. R. (2010). Common SNPs explain a large proportion of the heritability for human height. *Nat. Genet.* 42 565–569. 10.1038/ng.608 20562875 PMC3232052

[B41] Yazdi M. Visscher P. Ducrocq V. Thompson R. (2002). Heritability, reliability of genetic evaluations and response to selection in proportional hazard models. *J. Dairy Sci.* 85 1563–1577. 10.3168/jds.s0022-0302(02)74226-4 12146489

[B42] Yu X. Xiao L. Zeng P. Huang S. (2019). Jackknife model averaging prediction methods for complex phenotypes with gene expression levels by integrating external pathway information. computational and mathematical methods in medicine. *Comput. Math. Methods Med.* 2019:8. 10.1155/2019/2807470 PMC6476151 31089389

[B43] Zeng P. Zhou X. (2017). Non-parametric genetic prediction of complex traits with latent dirichlet process regression models. *Nat. Commun.* 8:456. 10.1038/s41467-017-00470-2 PMC5587666 28878256

[B44] Zeng P. Zhou X. Huang S. (2017). Prediction of gene expression with cis-SNPs using mixed models and regularization methods. *BMC Genom.* 18:368. 10.1186/s12864-017-3759-6 28490319 PMC5425981

[B45] Zhao H. Ljungberg B. Grankvist K. Rasmuson T. Tibshirani R. Brooks J. D. (2005). Gene expression profiling predicts survival in conventional renal cell carcinoma. *PLoS Med.* 3:e13. 10.1371/journal.pmed.0030013 16318415 PMC1298943

[B46] Zhao Q. Shi X. Xie Y. Huang J. Shia B. Ma S. (2014). Combining multidimensional genomic measurements for predicting cancer prognosis: observations from TCGA. *Brief. Bioinform.* 16 291–303. 10.1093/bib/bbu003 24632304 PMC4375393

[B47] Zhou J. Wu X. Li G. Gao X. Zhai M. Chen W. (2017). Prediction of radiosensitive patients with gastric cancer by developing gene signature. *Int. J. Oncol.* 51 1067–1076. 10.3892/ijo.2017.4107 28902346 PMC5592874

[B48] Zhou X. Carbonetto P. Stephens M. (2013). Polygenic modeling with bayesian sparse linear mixed models. *PLoS Genet.* 9:e1003264. 10.1371/journal.pgen.1003264 23408905 PMC3567190

[B49] Zhu B. Song N. Shen R. Arora A. Machiela M. J. Song L. (2017). Integrating clinical and multiple omics data for prognostic assessment across human cancers. *Sci. Rep.* 7:16954. 10.1038/s41598-017-17031-8 PMC5717223 29209073

[B50] Zou H. Hastie T. (2005). Regularization and variable selection via the elastic net. *J. Royal Stat. soc. Series B (statistical methodology)* 67 301–320. 10.1111/j.1467-9868.2005.00503.x

