# Supplementary material for: How Can Gene-Expression Information Improve Prognostic Prediction in TCGA Cancers: An Empirical Comparison Study on Regularization and Mixed Cox Models
Source: Front Genet. 2020 Aug 21;11:920. doi: 10.3389/fgene.2020.00920 (PMC7472843; doi:10.3389/fgene.2020.00920)
Supplement: Supplementary file 1 [file Data_Sheet_1.docx]

Supplemental Methods

### The general Cox model with only clinical covariates

Let ***X****_i_* be a *p*-dimensional vector for clinical covariates (e.g. disease stage, age and gender in the TCGA data ([Hoadley et al., 2018](#_ENREF_27)); see also Table 1) for individual *i*, and assume each of ***X*** is standardized to have mean zero and variance one. Denote the observed survival time by *t_i_* and the true survival time by *T_i_* with *d_i_*indicating the censored status; that is, *d_i_* = 1 if *T_i_* = *t_i_*, whereas *d_i_* = 0 if *t_i_* < *T_i_*. The sample size is *n*. To link the survival risk with the clinical information, we employ Cox proportional hazards regression ([Cox, 1972](#_ENREF_5)) as it is the most commonly used approach modeling censored survival data sets

$h(t_{i}|X_{i}) = h_{0}(t_{i})e^{X_{i}^{T}a}$,

where *h*_0_(*t*) is referred to as an arbitrary baseline hazard function corresponding to the reference level of covariates and can be interpreted as the hazard risk for individuals with ***X*** at the average level; ***a*** = (*a*_1_, *a*_2_,…, *a_p_*) is an *p*-dimensional vector of effect sizes for covariates. The log partial likelihood is ([Cox, 1975](#_ENREF_6))

$plog(a) =\sum_{i = 1}^{n} d_{i}\frac{e^{X_{i}^{T}a}}{\sum_{l\in R(t_{i})} e^{X_{l}^{T}a}}$,

where *R*(*t*) is the risk set at time *t*. If ties of the survival time are present, the log partial likelihood is suitably modified by the Breslow’s ([Breslow, 1975](#_ENREF_1)) or the Efron’s ([Efron, 1977](#_ENREF_14)) methods. The unknown parameters are estimated by maximizing the partial log-likelihood via the Newton-Raphson or cyclical coordinate descent algorithm ([Friedman et al., 2007](#_ENREF_18); [Friedman et al., 2010](#_ENREF_19)).

### Regularized Cox model with clinical covariates and gene expressions levels

Let ***G****_i_*be an *m*-dimensional vector for a set of genetic markers (e.g. expression levels for *m* genes in the TCGA data) for individual *i*, and assume each marker is standardized to have mean zero and variance one. The Cox model including both ***X****_i_* and ***G****_i_* is written as

$h(t_{i}|X_{i}, G_{i}) = h_{0}(t_{i})e^{X_{i}^{T}a + G_{i}^{T}b}$,

where ***b*** = (*b*_1_, *b*_2_, …, *b_m_*) is an *m*-dimensional vector of effect sizes for biomarkers. Denote the unknown parameters by ***θ*** = (***a***, ***b***). The log partial likelihood is

$plog(\theta) =\sum_{i = 1}^{n} d_{i}\frac{e^{X_{i}^{T}a + G_{i}^{T}b}}{\sum_{l\in R(t_{i})} e^{X_{l}^{T}a + G_{l}^{T}b}}$,

In our high-dimensional setting where the number of genes is much larger than the sample size (i.e. *m* >> *n*), the traditional maximum likelihood method for parameter estimation in model cannot be applicable. Alternatively, in the past few years a lot of regularization methods were developed ([Tibshirani, 1996b](#_ENREF_56); [Fan and Li, 2001](#_ENREF_16); [Zou and Hastie, 2005](#_ENREF_70); [Bühlmann and van de Geer, 2011](#_ENREF_2); [Hastie et al., 2015](#_ENREF_26)) and employed to estimate unknown parameters by imposing a penalty on the effect sizes of genes.

In the present study we focus on Lasso ([Tibshirani, 1996a](#_ENREF_55); [1997](#_ENREF_57)) and elastic net (ENET) ([Zou and Hastie, 2005](#_ENREF_70)) because they generally behave equally to or better than other regularization approaches in genetic prediction in terms of our prior experience ([Ping et al., 2017](#_ENREF_46)). Specifically, Lasso fits the following Cox model (denoted by coxlasso)

$$Q(\theta) = -plog(\theta) + \sum_{j =1}^{m} P_{\text{λ}}(b_{j}), P_{\text{λ}} = \text{λ}|b|,$$

and elastic net fits the following Cox model (denoted by coxenet)

$$Q(\theta) = -plog(\theta) + \sum_{j =1}^{m} P_{\text{λ}}(b_{j}), P_{\text{λ}} = \text{λ(}\alpha|b| + \text{(1} - \alpha\text{)}b^{2}),$$

where *P*_λ_ is the penalty function with λ indicating the turning parameter that controls for the extent of shrinkage, and *α* in elastic net provides a mix between ridge and Lasso ([Tibshirani, 1996b](#_ENREF_56); [Zou and Hastie, 2005](#_ENREF_70); [Hastie et al., 2009](#_ENREF_25); [Hastie et al., 2015](#_ENREF_26)). Due to the absolute penalty in coxlasso and coxenet, small effect sizes will be exactly shrunken to be zero with some reasonably selected value of λ, resulting in a sparse Cox model. It needs to empathize we do not impose any penalties on the effect sizes of clinical covariates and always include them into the model.

In our study we employ the coordinate descent algorithm ([Friedman et al., 2007](#_ENREF_18); [Friedman et al., 2010](#_ENREF_19)) to fit coxenet and select the optimal λ via 100-fold cross-validation ([Hastie et al., 2009](#_ENREF_25)). We set *α* = 0.5 as done in ([Gamazon et al., 2015](#_ENREF_20); [Ping et al., 2017](#_ENREF_46)) and employ the R glmnet (version 2.0-5) package ([Friedman et al., 2010](#_ENREF_19)).

### Linear mixed effects Cox model with clinical covariates and gene expressions levels

As mentioned above, the regularized methods (e.g. elastic net in ) are sparse models in nature, which assume only a few of genes are involved in the survival risk. Alternatively, we instead incorporate all the effect sizes of genes in the Cox model within the framework of linear mixed models ([De Los Campos et al., 2010](#_ENREF_9); [Yang et al., 2010](#_ENREF_61); [Makowsky et al., 2011](#_ENREF_42); [De los Campos et al., 2013a](#_ENREF_10); [De los Campos et al., 2013b](#_ENREF_11)). In contrast to elastic net, the Cox linear mixed model (denoted by coxlmm) can be viewed as a polygenic model in the sense that it explicitly assumes all genes can be implicated in the disease progress and have nonzero effects ([Zhou et al., 2013](#_ENREF_68); [Dandine-Roulland and Perdry, 2015](#_ENREF_8); [Ott, 2015](#_ENREF_45)). In the coxlmm model the effect size of the gene is supposed to follow a normal distribution

$h(t_{i}|X_{i}, G_{i}) = h_{0}(t_{i})e^{X_{i}^{T}a + G_{i}^{T}b}, \text{ }b_{j} \sim N(0, \text{σ}_{b}^{2})$,

where $\text{σ}_{b}^{2}$ is the variance for gene expressions and plays a key role to avoid overfitting in such a high-dimensional modeling.

Note that, for the mixed-effects Cox model constructed in , due to the second level of normal distribution for the effect sizes of expressions, we are actually modeling coxlmm within the framework of multi-level models or Bayesian hierarchical models ([Gelman and Hill, 2006](#_ENREF_22)). Furthermore, in terms of the similar penalty function shown in or , coxlmm can be also viewed as a penalty-type Cox model with the ridge regularization which has the square penalty on effect sizes rather than the obsolete penalty in Lasso. Ridge regression fits the following Cox model (denoted by coxridge)

$$Q(\theta) = -plog(\theta) + \sum_{j =1}^{m} P_{\text{λ}}(b_{j}), P_{\text{λ}} = \text{λ}b^{2},$$

The primary difference is that cross-validation is often used to determine the tuning parameter in ridge regressions, while the maximum likelihood algorithm is generally employed in mixed-effects models ([Hoerl and Kennard, 1970](#_ENREF_28)). Finally, we emphasize that both the two types of Cox models (i.e. the regularization approaches such as coxlasso, coxenet and coxridge, and the coxlmm method) can be interpreted from the Bayesian point of view. That is, the penalties in regularized Cox models and the second-level effect distribution in coxlmm can all be viewed as some kinds of prior distribution and the corresponding penalty likelihood or the joint likelihood can be viewed as the posterior distribution. Rather than using the general Bayesian posterior estimation algorithms (e.g. Gibbs sampling) to generate a distribution for each unknown parameter, in the present study we estimate all unknown parameters via frequentist approaches such as the coordinate descent algorithm and the maximum likelihood algorithm.

The coxlmm model is fitted with the R coxme (version 2.2-10) package ([Therneau, 2019](#_ENREF_53)), in which the Laplace approximation method is implemented based on the second order Taylor series ([Ripatti and Palmgren, 2000](#_ENREF_47); [Therneau et al., 2003](#_ENREF_54)). However, fitting coxlmm with coxme directly is time-consuming due to the high-dimensional problem. Instead, we fit coxlmm in an efficient alternative way. Specifically, note that the transcriptomic component $G_{i}^{T}b$ in model can be re-expressed as

$$u_{i} = G_{i}^{T}b \sim N(0, G_{i}^{T}G_{i}\text{σ}_{b}^{2}).$$

Based on the above relationship, we construct an equivalent Cox mixed model

$$h(t_{i}|X_{i}, G_{i}) = h_{0}(t_{i})e^{X_{i}^{T}a + Z_{i}^{T}\delta}, \delta_{j} \sim N(0, \text{σ}_{b}^{2}),$$

where **Z***_i_* is the *i*th row vector of **K**^1/2^ with **K** = **GG***^T^*, which is often referred to as the genetic relationship matrix in genetic prediction ([Yang et al., 2010](#_ENREF_61); [Makowsky et al., 2011](#_ENREF_42); [Zeng and Zhou, 2017](#_ENREF_64)); ***δ*** is an *n*-dimensional vector for the effect sizes of ***Z****_i_*. After the transformation, the dimension of the random effects reduces from *m* in to *n* in , leading to a much more efficient computation for coxlmm as *n* is typically much smaller than *m* in the TCGA data sets (Table 1). Finally, we estimate ***b*** by

$$\hat{b} = G^{T}K^{-1}\hat{u},$$

where $\hat{u}$ = $Z\hat{\delta}$ with $Z$ = **K**^1/2^ and $\hat{\delta}$ estimated from model via the Laplace approximation method based on the second order Taylor series expansion. It has been demonstrated that the Laplace approximation is very efficient to estimate the variance $\text{σ}_{b}^{2}$ both theoretically and empirically ([Ripatti and Palmgren, 2000](#_ENREF_47); [Therneau et al., 2003](#_ENREF_54)).

### Relative importance of clinical and transcriptomic components

We now quantify the relative overall importance of clinical and transcriptomic information (denoted by $X_{i}^{T}a$ and $G_{i}^{T}b$, respectively) for survival phenotypes. However, unlike the case of linear mixed models for continuous phenotypes ([Visscher et al., 2008](#_ENREF_58); [Yang et al., 2010](#_ENREF_61); [Lee et al., 2011](#_ENREF_36); [Speed et al., 2012](#_ENREF_52); [Yang et al., 2017](#_ENREF_62); [Hou et al., 2019](#_ENREF_29); [Speed and Balding, 2019](#_ENREF_51)), it is not trivial to define the relative importance in the Cox model. To solve this problem, we first apply *F*(*t_i_*) and *S*(*t_i_*) to denote the cumulative distribution function and the survival function of the survival time, respectively. Then, we have

$$F(t_{i}) = 1 - S(t_{i}) = 1 - e^{-H(t_{i})}\text{,}$$

with *H*(*t_i_*) the cumulative hazard function of the hazard risk

$$H(t_{i}) = \int_{0}^{t_{i}} h(s)ds = H_{0}(t_{i})e^{X_{i}^{T}a + G_{i}^{T}b}.$$

Note that, *F*(*t_i_*) is uniformly distributed between 0 and 1, and *H*(*t_i_*) is exponentially distributed with parameter 1. A log-transformation on *H*(*t_i_*) leads to

$$e_{i} = \log\{H(t_{i})\} = \log\{H_{0}(t_{i})\} + X_{i}^{T}a + G_{i}^{T}b$$

which follows a standard extreme value distribution with variance *π*^2^/6. Re-arranging the terms in , we have

$\log\{H_{0}(t_{i})\} = - X_{i}^{T}a - G_{i}^{T}b + e_{i}$.

This transformation provides a feasible manner to quantify the relative overall importance of covariates for survival phenotypes ([Ducrocq and Casella, 1996](#_ENREF_13); [Korsgaard et al., 1998](#_ENREF_34); [Korsgaard et al., 1999](#_ENREF_33); [Yazdi et al., 2002](#_ENREF_63); [Gorfine et al., 2017](#_ENREF_23)). We thus consider the following two quantities

$$\begin{matrix} \text{PCE}=\theta_{c}=\frac{\text{var(}Xa\text{)}}{\text{var(}Xa\text{)} + \text{var(}Gb\text{)} + \text{var(}e\text{)}}, \\ \text{PGE}=\theta_{g}=\frac{\text{var(}Gb\text{)}}{\text{var(}Xa\text{)} + \text{var(}Gb\text{)} + \text{var(}e\text{)}}, \end{matrix}$$

where var(*x*) is the variance of *x* and calculated by $E(x-E(x)^{2})$/*n* with *E*(*x*) the expectation of *x*, and var(***e***) represents all the remaining unexplained environment components except ***X*** and ***G***. The definitions above ensure that both PCE and PGE are bounded between 0 and 1, sharing the similar interpretation of heritability ([Korsgaard et al., 1999](#_ENREF_33)). PCE reveals how much the survival variation can be explained with only the clinical information; while PGE reveals how much the survival variation can be explained with only the transcriptomic information. The summation of PCE and PGE can be viewed as the proportion of the survival variance explained (PVE) by currently available clinical and transcriptomic information together in the Cox model.

We empirically calculate PCE and PGE on the scale of the transformed hazard function shown in once ***a*** and ***b*** are estimated with coxenet or coxlmm. In order to yield confidence intervals for PCE or PGE, we apply the Jackknife method ([Efron and Tibshirani, 1994](#_ENREF_15)). For each jackknife sample, we yield the jackknife estimate $\hat{\theta}_{i}$ for *θ_c_* or *θ_g_*. As these quantities are limited between 0 and 1, we perform logit transformation for these jackknife estimates with $\text{logit}(\hat{\theta}_{i})=\ln(\hat{\theta}_{i}/(1-\hat{\theta}_{i}))$. A bias-corrected transformation of $\hat{\theta}_{i}$ is further implemented in terms of the pseudo-values method

$\tilde{\theta}_{i} = n\text{logit}(\hat{\theta}) - (n - 1)\text{logit}(\hat{\theta}_{i})$,

where $\hat{\theta}$ is the original estimate of PCE or PGE with the total samples. The mean of the jackknife estimate is $\bar{\theta} = \sum_{i = 1}^{n} \tilde{\theta}_{i}/n$, with the standard error

$\text{se}(\tilde{\theta}) = \frac{1}{(n - 1)n}\sum_{i = 1}^{n} (\tilde{\theta}_{i} - \bar{\theta})^{2}$.

The 95% confidence intervals for the transformed jackknife estimate are calculated as

$$\bar{\theta} \pm1.96 \times\sqrt{\text{se}(\tilde{\theta})}$$

Finally, the 95% confidence intervals for PCE or PGE can be easily obtained by using the inverse logit transformation.

Supplemental Results

Table S1. A selective overview of previous studies on prediction using omics information

| **Id** | **Year** | **Cancer** | **Type of omics data** | **Number of signatures** | **Conclusion** | **Reference** |
| --- | --- | --- | --- | --- | --- | --- |
| 1 | 2019 | Early-stage lung adenocarcinoma | DNA methylation and RNA (mRNA) expression | 12 DNA methylation probes and 7 overlapping genes | The model performed better than all existing models for early-stage LUAD | ([Dong et al., 2019](#_ENREF_12)) |
| 2 | 2019 | Pancreatic carcinoma | RNA (mRNA) expression | 7 genes | The seven genes/proteins-based model can predict the survival of PC valuably and robustly | ([Cheng et al., 2019](#_ENREF_4)) |
| 3 | 2019 | Hepatocellular carcinoma | RNA (mRNA) expression; RNA (miRNA) expression and DNA methylation | 37 top features | The multi-omics model had a great performance on five external datasets of various omics types | ([Chaudhary et al., 2018](#_ENREF_3)) |
| 4 | 2019 | Gastric cancer | RNA (mRNA) expression | 13 mRNAs | The 13 mRNA-based risk score model could distinguish the prognosis and predict the survival of GC patients well in both TCGA and GEO datasets | ([Dai et al., 2019](#_ENREF_7)) |
| 5 | 2019 | Breast cancer | RNA (miRNA) expression | 6-miRNA signature | The six-miRNA-based model was able to predict the prognosis of BC patients reliably. | ([Lai et al., 2019](#_ENREF_35)) |
| 6 | 2019 | hepatocellular carcinoma | RNA (mRNA) expression | 4-gene-metabolic signature | The four-gene metabolic signatures were novel and robust on the HCC prognosis prediction. | ([Liu et al., 2019](#_ENREF_40)) |
| 7 | 2018 | Adrenocortical carcinoma | RNA (mRNA) expression | 5 genes | The five-gene-based model played an important role in the prediction of ACC prognosis | ([Xiao et al., 2018](#_ENREF_60)) |
| 8 | 2018 | Oral squamous cell carcinoma | RNA (mRNA) expression | 3 genes | Three mRNA expression signatures were identified to have a great prognostic prediction. | ([Zhao et al., 2018](#_ENREF_66)) |
| 9 | 2018 | Endometrial carcinoma | RNA (mRNA) expression | 1 gene | TFAP2B mRNA expression may play a potential role in prognostic prediction of endometrial cancer | ([Wu and Zhang, 2018](#_ENREF_59)) |
| 10 | 2018 | Non‐small cell lung cancer | RNA (lncRNA) expression | 7 lncRNA | A model including seven‐lncRNA signatures can well predict survival of NSCLC patients | ([Lin et al., 2018](#_ENREF_39)) |
| 11 | 2018 | Lower grade glioma | RNA (mRNA) expression | 31-gene signature | The 31-gene radio-sensitivity signature and PD-L1 expression status were identified to be important factors in prediction of the LGG patients | ([Jang and Kim, 2018](#_ENREF_31)) |
| 12 | 2018 | Hepatocellular carcinoma | RNA (mRNA) expression | 4 genes | The four‐mRNA prognostic model can well predict the OS of HCC patients | ([Long et al., 2018](#_ENREF_41)) |
| 13 | 2018 | Ovarian serous cystadenocarcinoma | DNA methylation | 5 DNA methylation | This five-DNA methylation signature was identified to be a novel prognostic indicator and utilized to predict the prognosis of Ovarian serous cystadenocarcinoma patients | ([Guo et al., 2018](#_ENREF_24)) |
| 14 | 2018 | Glioblastoma multiform | The protein coding genes (PCGs) and RNA (lncRNA) expression | 6 PCGs and 5 lncRNA | The PCG-lncRNA signature was identified to be a novel prognostic marker to predict prognosis of GBM patients after surgery | ([Gao et al., 2018](#_ENREF_21)) |
| 15 | 2018 | Breast cancer | RNA (miRNA) expression | 3 miRNAs | The novel miRNA-based risk score was developed for prediction of survival and recurrence potential in breast cancer. | ([Kawaguchi et al., 2018](#_ENREF_32)) |
| 16 | 2018 | Pancreatic Adenocarcinoma | RNA (miRNA) expression | 5 microRNA | The five-miRNA signature could be used to predict the prognostic of pancreatic adenocarcinoma well | ([Shi et al., 2018](#_ENREF_50)) |
| 17 | 2018 | Cervical squamous cell carcinoma | RNA (lncRNA) expression | 15-lncRNA expression signature | A 15-lncRNA expression signature can well predict survival time of cervical cancer patient. | ([Mao et al., 2018a](#_ENREF_43)) |
| 18 | 2018 | Esophageal squamous cell carcinoma | lncRNA expression profiles | 7-lncRNA signature | This seven-lncRNA signature model could predict prognosis of patients with ESCC | ([Mao et al., 2018b](#_ENREF_44)) |
| 19 | 2018 | Hepatocellular carcinoma | DNA methylation and RNA (mRNA) expression | 5 CpG sites | The integrated prognostic signature model could improve the survival prediction in Hepatocellular carcinoma | ([Fang et al., 2018](#_ENREF_17)) |
| 20 | 2017 | oral squamous cell carcinoma | DNA methylation and RNA (mRNA) expression | 7 DNA methylation CpG sites | Prognostic signature integrated of DNA methylation, gene expression, and clinical information could improve the prognostic prediction accuracy of model only with clinical information for OSCC patients | ([Shen et al., 2017b](#_ENREF_49)) |
| 21 | 2017 | Gastric cancer | RNA (mRNA) expression | Top 11 significant genes | These genes could play a potential role in diagnosis and prediction of gastric cancer | ([Zhou et al., 2017](#_ENREF_67)) |
| 22 | 2017 | Head and neck squamous cell carcinoma | RNA (mRNA) expression | 7 genes | The seven-gene prognostic signature could predict the prognosis of HNSCC patients reliably | ([Shen et al., 2017a](#_ENREF_48)) |
| 23 | 2017 | Breast cancer | RNA (mRNA) expression | 31-gene signature | The 31 radio-sensitivity gene signature and PD-L1 status were identified to play an important role in the prediction of clinical outcome in patients with breast cancer | ([Jang and Kim, 2017](#_ENREF_30)) |
| 24 | 2017 | Squamous cell lung carcinoma | RNA (mRNA) expression | 4 gene clustering model in 14 genes | This model would do well in prediction of patients’ outcomes with SQCLC and then improve the treatment strategies | ([Li et al., 2017](#_ENREF_37)) |
| 25 | 2014 | Breast invasive carcinoma, Glioblastoma multiforme, Acute myeloid leukemia and Lung squamous cell carcinoma | RNA (mRNA) expression, DNA methylation, RNA (miRNA) expression and copy number alterations | PCA, PLS and Lasso | The mRNA-gene expression may have the best predictive power, and there is no significant gain by integrating other types of omics data | ([Zhao et al., 2014](#_ENREF_65)) |
| 26 | 2014 | Breast cancer | RNA (miRNA) expression | 14 miRNAs | A miRNA signature including 14 ER-related miRNAs could perform well in prognostic prediction of ER-positive breast cancer | ([Zhou et al., 2014](#_ENREF_69)) |
| 27 | 2014 | Lung adenocarcinoma | RNA (miRNA) expression | 8-miRNA signature | The miRNA signature comprising eight miRNAs could independently predict prognostic of LUAD patient survival | ([Li et al., 2014](#_ENREF_38)) |

Table S2. Comparison of predciton performance for 32 TCGA cancers

| **Cancer** | **cox** | **coxenet** | **coxlasso** | **coxlmm** | |
| --- | --- | --- | --- | --- | --- |
|  |  |  |  | **original** | **permuted** |
| ACC | 0.75±0.11 | 0.80±0.09 | 0.79±0.10 | 0.83±0.09 | 0.74±0.11 |
| BLCA | 0.65±0.04 | 0.66±0.04 | 0.66±0.04 | 0.69±0.04 | 0.65±0.04 |
| BRCA | 0.74±0.04 | 0.74±0.05 | 0.74±0.05 | 0.75±0.05 | 0.74±0.05 |
| CESC | 0.63±0.09 | 0.66±0.10 | 0.65±0.10 | 0.72±0.09 | 0.63±0.09 |
| CHOL | 0.49±0.17 | 0.49±0.17 | 0.48±0.17 | 0.49±0.17 | 0.49±0.16 |
| COAD | 0.71±0.08 | 0.71±0.08 | 0.71±0.08 | 0.71±0.08 | 0.71±0.08 |
| DLBC | 0.54±0.30 | 0.53±0.30 | 0.54±0.30 | 0.53±0.30 | 0.51±0.31 |
| ESCA | 0.64±0.09 | 0.64±0.09 | 0.64±0.09 | 0.64±0.09 | 0.64±0.09 |
| GBM | 0.61±0.07 | 0.60±0.07 | 0.60±0.07 | 0.61±0.06 | 0.60±0.07 |
| HNSC | 0.59±0.05 | 0.63±0.04 | 0.63±0.04 | 0.65±0.05 | 0.59±0.05 |
| KICH | 0.89±0.12 | 0.89±0.12 | 0.89±0.12 | 0.88±0.13 | 0.89±0.12 |
| KIRC | 0.75±0.04 | 0.78±0.03 | 0.78±0.03 | 0.78±0.03 | 0.75±0.04 |
| KIRP | 0.81±0.10 | 0.84±0.09 | 0.83±0.10 | 0.86±0.07 | 0.81±0.09 |
| LAML | 0.68±0.06 | 0.69±0.06 | 0.68±0.06 | 0.70±0.06 | 0.68±0.06 |
| LGG | 0.79±0.05 | 0.85±0.04 | 0.85±0.04 | 0.86±0.03 | 0.79±0.05 |
| LIHC | 0.63±0.06 | 0.64±0.06 | 0.63±0.06 | 0.68±0.06 | 0.62±0.06 |
| LUAD | 0.67±0.05 | 0.69±0.05 | 0.69±0.05 | 0.70±0.05 | 0.67±0.05 |
| LUSC | 0.56±0.05 | 0.57±0.05 | 0.57±0.05 | 0.57±0.05 | 0.56±0.05 |
| MESO | 0.48±0.10 | 0.67±0.09 | 0.65±0.10 | 0.72±0.08 | 0.47±0.10 |
| OV | 0.62±0.05 | 0.62±0.05 | 0.62±0.05 | 0.61±0.05 | 0.61±0.05 |
| PAAD | 0.54±0.07 | 0.58±0.07 | 0.57±0.07 | 0.62±0.07 | 0.54±0.07 |
| PCPG | 0.69±0.25 | 0.68±0.25 | 0.67±0.25 | 0.72±0.22 | 0.64±0.25 |
| PRAD | 0.46±0.28 | 0.45±0.27 | 0.46±0.28 | 0.40±0.22 | 0.43±0.26 |
| READ | 0.76±0.16 | 0.76±0.15 | 0.76±0.16 | 0.77±0.16 | 0.76±0.16 |
| SARC | 0.57±0.08 | 0.66±0.07 | 0.65±0.07 | 0.70±0.06 | 0.56±0.08 |
| SKCM | 0.63±0.05 | 0.68±0.05 | 0.55±0.05 | 0.69±0.04 | 0.63±0.05 |
| STAD | 0.64±0.05 | 0.65±0.05 | 0.65±0.04 | 0.67±0.05 | 0.64±0.05 |
| THCA | 0.92±0.06 | 0.92±0.06 | 0.92±0.06 | 0.92±0.06 | 0.92±0.07 |
| THYM | 0.69±0.20 | 0.69±0.20 | 0.68±0.20 | 0.70±0.17 | 0.68±0.20 |
| UCEC | 0.78±0.10 | 0.78±0.10 | 0.79±0.09 | 0.77±0.10 | 0.78±0.10 |
| UCS | 0.66±0.14 | 0.65±0.14 | 0.65±0.14 | 0.64±0.15 | 0.65±0.14 |
| UVM | 0.67±0.16 | 0.76±0.15 | 0.75±0.14 | 0.80±0.14 | 0.67±0.15 |

Note: Cox denotes the Cox model with clinical information alone; coxlasso denotes the Cox model with clinical and gene expression information and estimated with lasso penalty; coxenet denotes the Cox model with clinical and gene expression information and estimated with elastic net penalty; coxlmm denote the Cox model with clinical and gene expression information and estimated within the framework of mixed model.

Table S3. Estimates and confidence intervals of PCE and PGE for 32 TCGA cancer data sets

| **Cancer** | **PCE** | **(95% CI)** | **PGE** | **(95% CI)** |
| --- | --- | --- | --- | --- |
| ACC | 21.54 | (6.71,51.73) | 41.12 | (12.71,76.53) |
| BLCA | 14.95 | (8.76,24.35) | 10.88 | (3.98,26.42) |
| BRCA | 22.48 | (14.46,33.23) | 13.43 | (4.27,34.99) |
| CESC | 12.28 | (5.34,25.81) | 24.38 | (10.87,45.91) |
| CHOL | 7.80 | (0.53,58.30) | 0.00 | (0.00, 0.00) |
| COAD | 31.77 | (19.01,48.02) | 4.53 | (0.07,75.73) |
| DLBC | 21.32 | (1.85,80.34) | 0.00 | (0.00, 0.00) |
| ESCA | 21.10 | (10.79,37.21) | 0.00 | (0.00, 0.00) |
| GBM | 9.03 | (3.07,23.77) | 8.61 | (0.65,57.03) |
| HNSC | 10.10 | (5.09,19.06) | 12.43 | (5,27.61) |
| KICH | 71.72 | (47.35,87.81) | 0.21 | (0.00,100) |
| KIRC | 25.71 | (18.01,35.29) | 14.13 | (7.73,24.42) |
| KIRP | 27.50 | (12.02,51.41) | 30.25 | (13.73,54.04) |
| LAML | 22.52 | (12.76,36.64) | 19.66 | (6.56,45.82) |
| LGG | 23.87 | (14.95,35.89) | 39.13 | (26.4,53.49) |
| LIHC | 8.57 | (4.24,16.57) | 12.25 | (4.21,30.64) |
| LUAD | 11.02 | (6.51,18.07) | 12.39 | (5,27.53) |
| LUSC | 4.29 | (1.68,10.53) | 0.31 | (0,28.43) |
| MESO | 2.39 | (0.31,16.21) | 62.10 | (32.42,84.66) |
| OV | 6.65 | (2.39,17.16) | 3.93 | (0.02,88.73) |
| PAAD | 4.02 | (1.03,14.49) | 21.78 | (7.82,47.74) |
| PCPG | 34.17 | (2.29,91.4) | 54.29 | (1.66,98.7) |
| PRAD | 2.16 | (0.00,100) | 9.81 | (0.00,100) |
| READ | 55.25 | (24.59,82.39) | 8.33 | (0.00,100) |
| SARC | 5.37 | (1.56,16.93) | 35.25 | (17.09,58.84) |
| SKCM | 10.29 | (5.83,17.56) | 16.56 | (8.13,30.81) |
| STAD | 18.21 | (10.86,28.91) | 5.59 | (1.20,22.3) |
| THCA | 69.62 | (54.74,81.3) | 0.00 | (0.00,100) |
| THYM | 26.99 | (5.95,68.54) | 17.23 | (0.15,96.7) |
| UCEC | 34.52 | (17.37,56.98) | 0.52 | (0,99.95) |
| UCS | 17.55 | (4.89,47.02) | 0.00 | (0.00,27.41) |
| UVM | 20.76 | (6.45,50.5) | 46.85 | (23.03,71.83) |


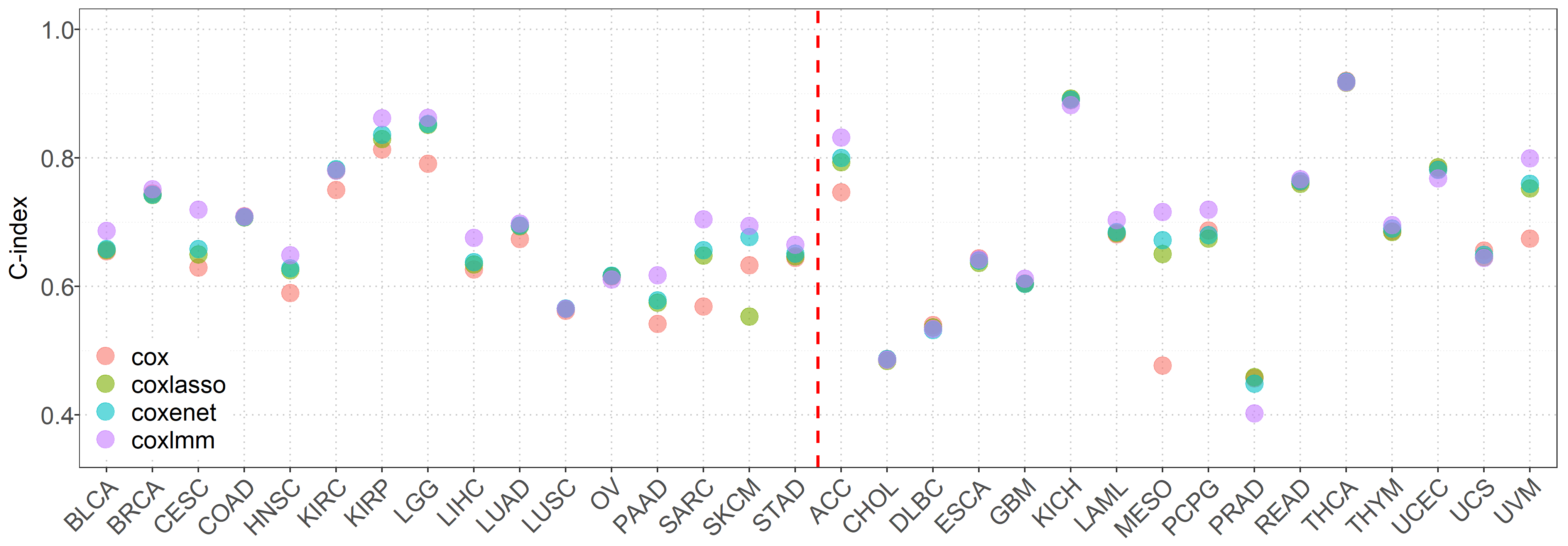


Figure S1. Performance compasion of four prediction models for the 32 TCGA cancers. Here cox denotes the general Cox model with only clinical covariates. The cancers located in the left of the red dotted line are low-censored while the cancers in the right side are high-censored.


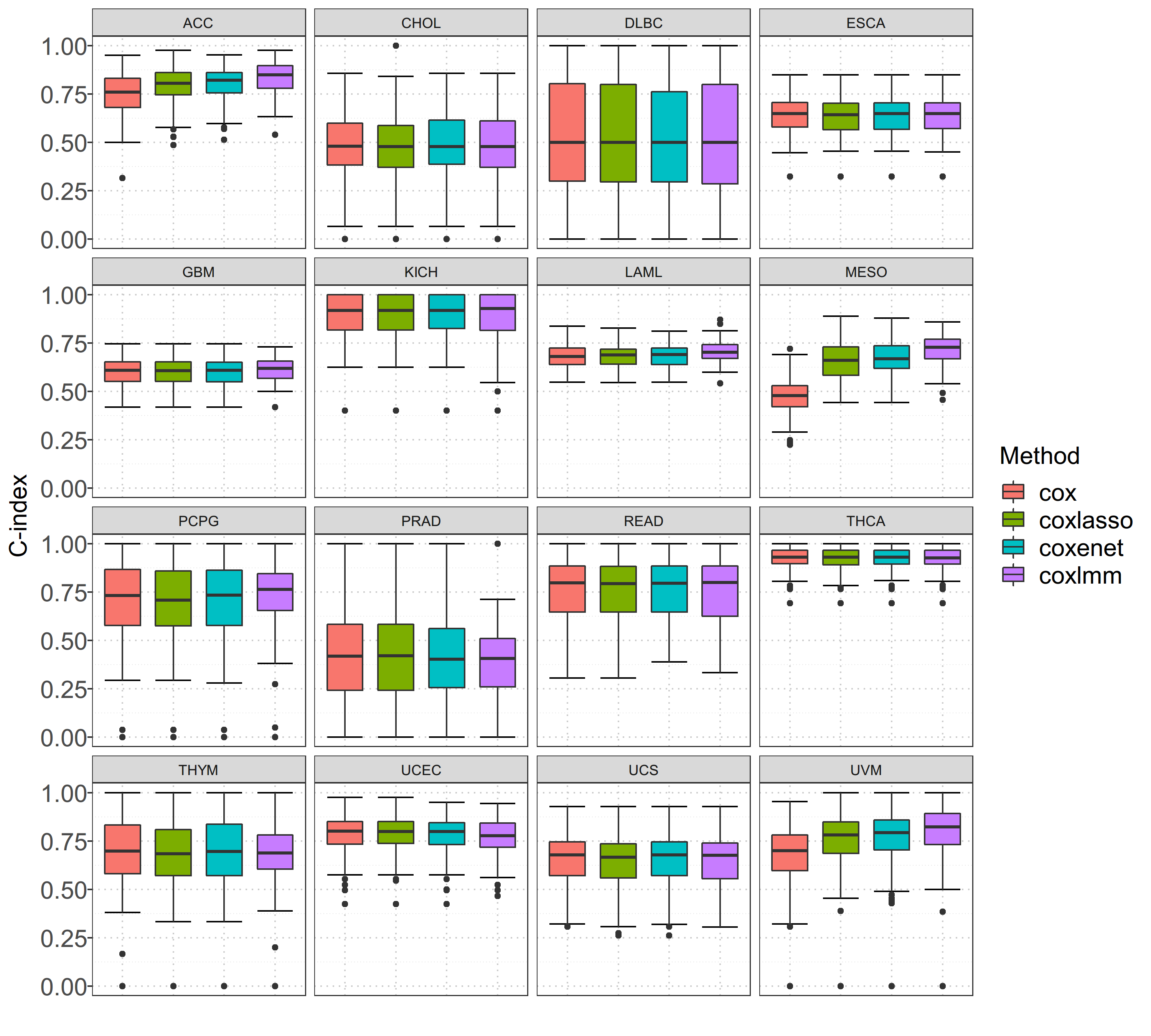


Figure S2. Comparison of predictive performance of four models in 16 high-censored cancers. Performance is measured by C-index and the predictive performance was assessed across 100 replicates.


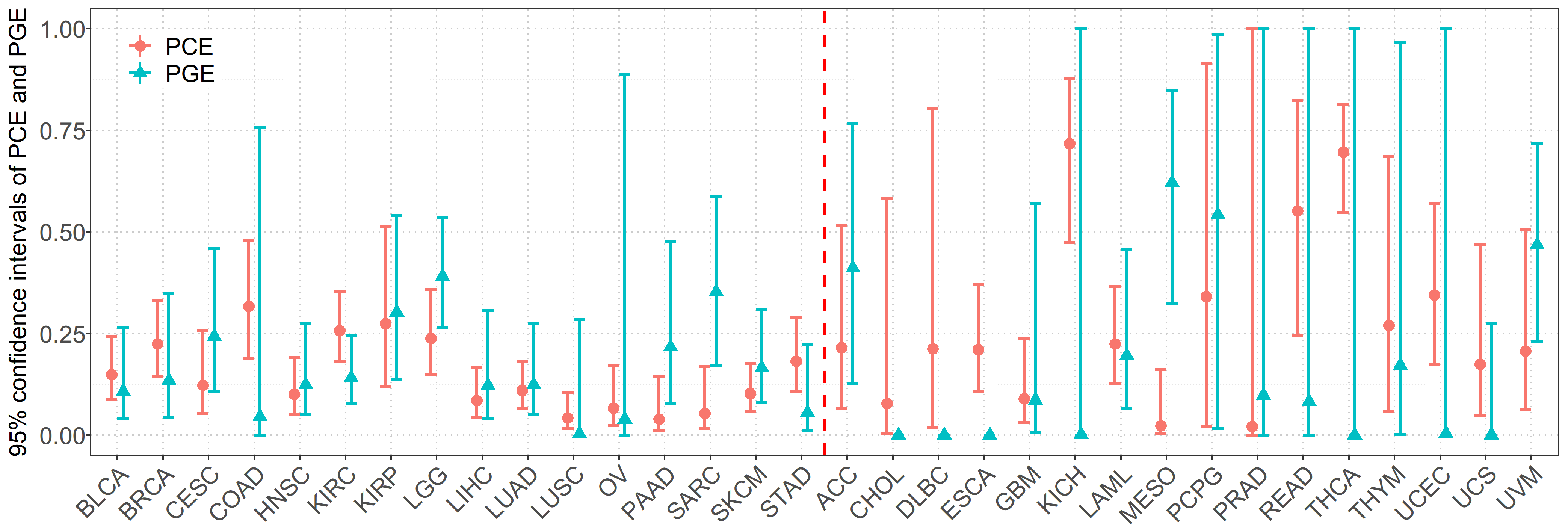


Figure S3. Point estimates and 95% confidence intervals of PCE and PGE for the 32 TCGA cancers. The cancers located in the left of the red dotted line are low-censored while the cancers in the right side are high-censored. PCE represents the proportion of the survival variation explained by the clinical information alone. PGE represents the proportion of the survival variation explained by the transcriptome information alone.


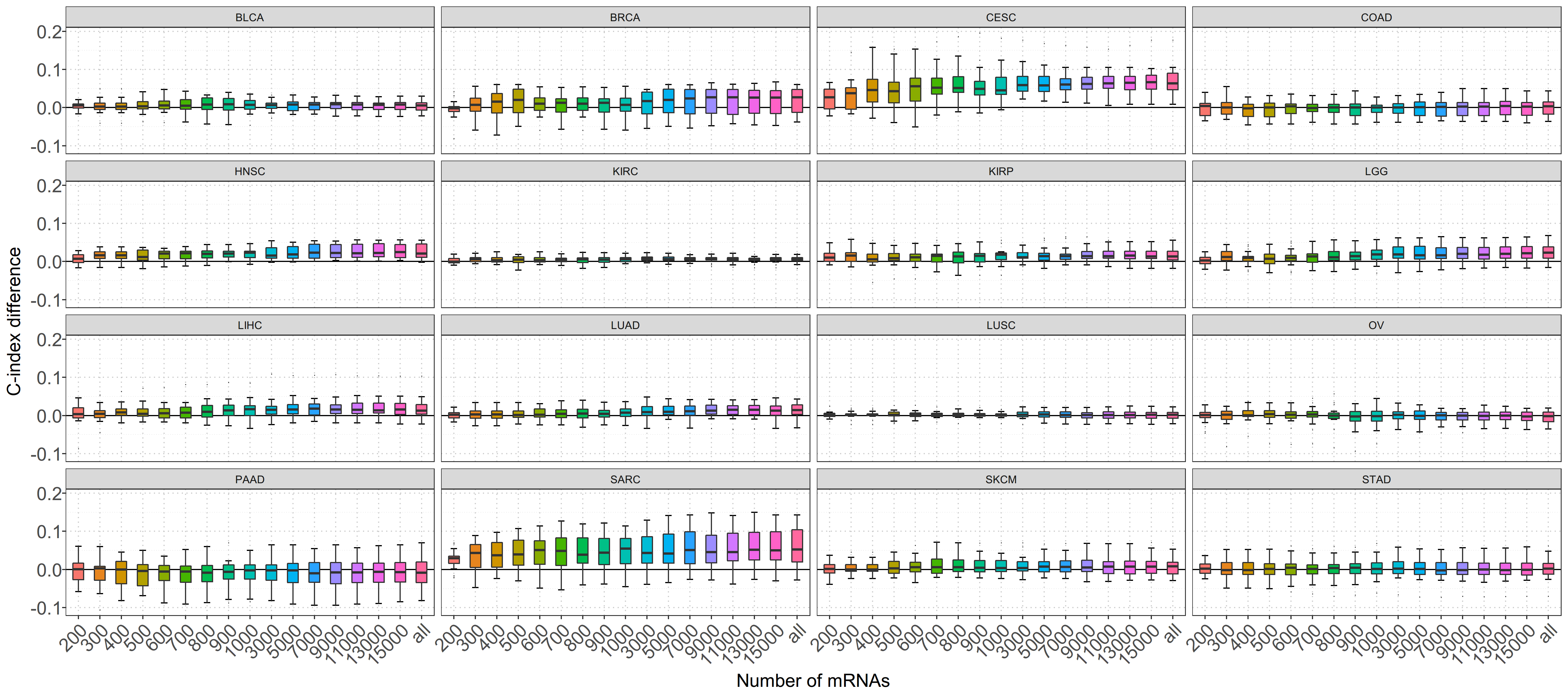


Figure S4. Comparison of predictive performance of coxlmm integrating different number of genes with 100 genes in 16 low-censored cancers. Performance is measured by C-index difference with respect to model with 100 genes, therefore, a negative value (i.e. values below the horizontal line) indicates worse performance than model with 100 genes. The predictive performance was assessed across 20 replicates.


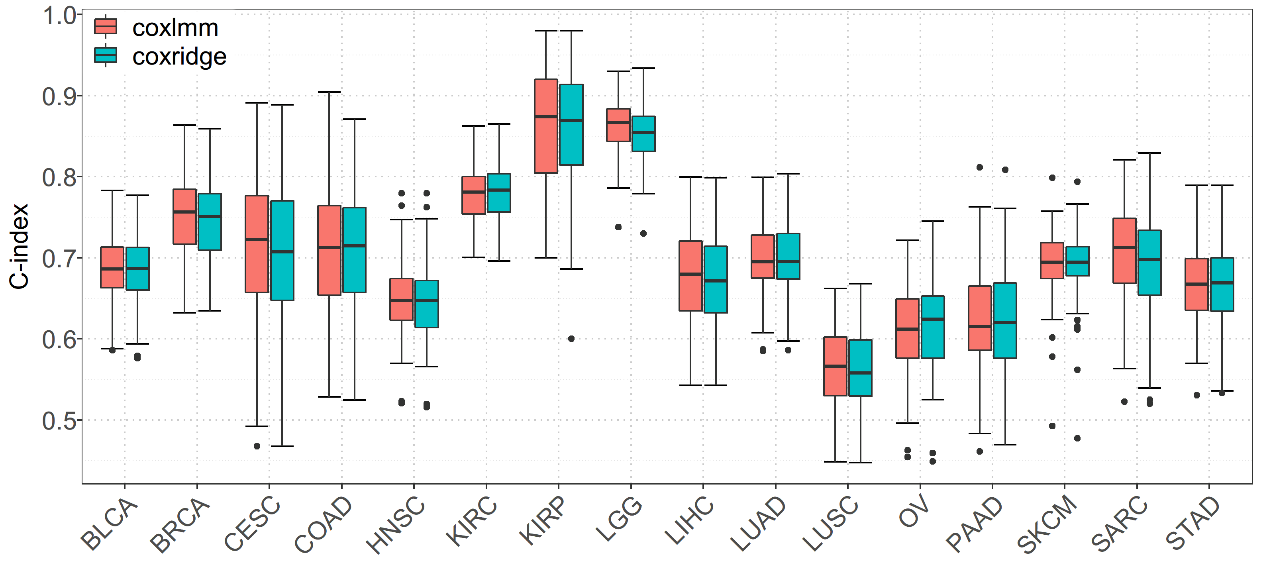


Figure S5. Comparison of predictive performance of coxlmm and coxridge in 16 low-censored cancers. Performance is measured by C-index and the predictive performance was assessed across 100 replicates.

**References**

Breslow, N.E. (1975). Analysis of Survival Data under the Proportional Hazards Model. *International Statistical Review / Revue Internationale de Statistique* 43(1)**,** 45-57. doi: 10.2307/1402659.

Bühlmann, P., and van de Geer, S. (2011). *Statistics for High-Dimensional Data: Methods, Theory and Applications.* Berlin: Springer Verlag.

Chaudhary, K., Poirion, O.B., Lu, L., and Garmire, L.X. (2018). Deep learning–based multi-omics integration robustly predicts survival in liver cancer. *Clinical Cancer Research* 24(6)**,** 1248-1259.

Cheng, Y., Wang, K., Geng, L., Sun, J., Xu, W., Liu, D., et al. (2019). Identification of candidate diagnostic and prognostic biomarkers for pancreatic carcinoma. *EBioMedicine* 40**,** 382-393.

Cox, D.R. (1972). Regression Models and Life-Tables. *Journal of the royal statistical society. Series B (Methodological)* 34(2)**,** 187-220.

Cox, D.R. (1975). Partial likelihood. *Biometrika* 62(2)**,** 269-276. doi: 10.1093/biomet/62.2.269.

Dai, J., Li, Z.-X., Zhang, Y., Ma, J.-L., Zhou, T., You, W.-C., et al. (2019). Whole Genome Messenger RNA Profiling Identifies a Novel Signature to Predict Gastric Cancer Survival. *Clinical and translational gastroenterology* 10(1)**,** 1.

Dandine-Roulland, C., and Perdry, H. (2015). The use of the linear mixed model in human genetics. *Human heredity* 80(4)**,** 196-206.

De Los Campos, G., Gianola, D., and Allison, D.B. (2010). Predicting genetic predisposition in humans: the promise of whole-genome markers. *Nature Reviews Genetics* 11(12)**,** 880.

De los Campos, G., Hickey, J.M., Pong-Wong, R., Daetwyler, H.D., and Calus, M.P. (2013a). Whole-genome regression and prediction methods applied to plant and animal breeding. *Genetics* 193(2)**,** 327-345.

De los Campos, G., Vazquez, A.I., Fernando, R., Klimentidis, Y.C., and Sorensen, D. (2013b). Prediction of complex human traits using the genomic best linear unbiased predictor. *PLoS genetics* 9(7)**,** e1003608.

Dong, X., Zhang, R., He, J., Lai, L., Alolga, R.N., Shen, S., et al. (2019). Trans-omics biomarker model improves prognostic prediction accuracy for early-stage lung adenocarcinoma. *Aging (Albany NY)* 11(16)**,** 6312.

Ducrocq, V., and Casella, G. (1996). A Bayesian analysis of mixed survival models. *Genetics, Selection, Evolution : GSE* 28(6)**,** 505-529. doi: 10.1186/1297-9686-28-6-505.

Efron, B. (1977). The efficiency of Cox's likelihood function for censored data. *Journal of the American statistical Association* 72(359)**,** 557-565.

Efron, B., and Tibshirani, R.J. (1994). *An introduction to the bootstrap.* CRC press.

Fan, J., and Li, R. (2001). Variable selection via nonconcave penalized likelihood and its oracle properties. *Journal of American Statistical Association* 96(456)**,** 1348-1360. doi: 10.2307/3085904.

Fang, F., Wang, X., and Song, T. (2018). Five-CpG-based prognostic signature for predicting survival in hepatocellular carcinoma patients. *Cancer biology & medicine* 15(4)**,** 425.

Friedman, J., Hastie, T., Höfling, H., and Tibshirani, R. (2007). Pathwise coordinate optimization. *The annals of applied statistics* 1(2)**,** 302-332.

Friedman, J., Hastie, T., and Tibshirani, R. (2010). Regularization paths for generalized linear models via coordinate descent. *Journal of statistical software* 33(1)**,** 1.

Gamazon, E.R., Wheeler, H.E., Shah, K.P., Mozaffari, S.V., Aquino-Michaels, K., Carroll, R.J., et al. (2015). A gene-based association method for mapping traits using reference transcriptome data. *Nature genetics* 47(9)**,** 1091.

Gao, W.-Z., Guo, L.-M., Xu, T.-Q., Yin, Y.-H., and Jia, F. (2018). Identification of a multidimensional transcriptome signature for survival prediction of postoperative glioblastoma multiforme patients. *Journal of translational medicine* 16(1)**,** 368.

Gelman, A., and Hill, J. (2006). *Data analysis using regression and multilevel/hierarchical models.* Cambridge university press.

Gorfine, M., Berndt, S.I., Chang-Claude, J., Hoffmeister, M., Le Marchand, L., Potter, J., et al. (2017). Heritability Estimation using a Regularized Regression Approach (HERRA): Applicable to continuous, dichotomous or age-at-onset outcome. *PLoS ONE* 12(8)**,** e0181269. doi: 10.1371/journal.pone.0181269.

Guo, W., Zhu, L., Yu, M., Zhu, R., Chen, Q., and Wang, Q. (2018). A five-DNA methylation signature act as a novel prognostic biomarker in patients with ovarian serous cystadenocarcinoma. *Clinical epigenetics* 10(1)**,** 142.

Hastie, T., Tibshirani, R., and Friedman, J. (2009). *The Elements of Statistical learning: Data Mining, Inference, and Prediction.* New York: Springer.

Hastie, T., Tibshirani, R., and Wainwright, M. (2015). *Statistical learning with sparsity: the lasso and generalizations.* New York: CRC Press.

Hoadley, K.A., Yau, C., Hinoue, T., Wolf, D.M., Lazar, A.J., Drill, E., et al. (2018). Cell-of-Origin Patterns Dominate the Molecular Classification of 10,000 Tumors from 33 Types of Cancer. *Cell* 173(2)**,** 291-304.e296. doi: 10.1016/j.cell.2018.03.022.

Hoerl, A.E., and Kennard, R.W. (1970). Ridge regression: Biased estimation for nonorthogonal problems. *Technometrics* 12(1)**,** 55-67.

Hou, K., Burch, K.S., Majumdar, A., Shi, H., Mancuso, N., Wu, Y., et al. (2019). Accurate estimation of SNP-heritability from biobank-scale data irrespective of genetic architecture. *bioRxiv***,** 526855.

Jang, B.-S., and Kim, I.A. (2017). A radiosensitivity gene signature and PD-L1 status predict clinical outcome of patients with invasive breast carcinoma in The Cancer Genome Atlas (TCGA) dataset. *Radiotherapy and Oncology* 124(3)**,** 403-410.

Jang, B.-S., and Kim, I.A. (2018). A radiosensitivity gene signature and PD-L1 predict the clinical outcomes of patients with lower grade glioma in TCGA. *Radiotherapy and Oncology* 128(2)**,** 245-253.

Kawaguchi, T., Yan, L., Qi, Q., Peng, X., Edge, S.B., Young, J., et al. (2018). Novel MicroRNA-based risk score identified by integrated analyses to predict metastasis and poor prognosis in breast cancer. *Annals of surgical oncology* 25(13)**,** 4037-4046.

Korsgaard, I.R., Andersen, A.H., and Jensen, J. (1999). Discussion of heritability of survival traits. *Page 31 in Proc. Int. Workshop on Genetic Improvement of Functional Traits in cattle, Longevity, Jouy-en-Josas, France. INTERBULL Bull. No. 21. Int. Bull Eval. Serv., Uppsala, Sweden*.

Korsgaard, I.R., Madsen, P., and Jensen, J. (1998). Bayesian inference in the semiparametric log normal frailty model using Gibbs sampling. *Genetics Selection Evolution* 30(3)**,** 241.

Lai, J., Wang, H., Pan, Z., and Su, F. (2019). A novel six-microRNA-based model to improve prognosis prediction of breast cancer. *Aging (Albany NY)* 11(2)**,** 649.

Lee, S.H., Wray, N.R., Goddard, M.E., and Visscher, P.M. (2011). Estimating missing heritability for disease from genome-wide association studies. *The American Journal of Human Genetics* 88(3)**,** 294-305.

Li, J., Wang, J., Chen, Y., Yang, L., and Chen, S. (2017). A prognostic 4‐gene expression signature for squamous cell lung carcinoma. *Journal of cellular physiology* 232(12)**,** 3702-3713.

Li, X., Shi, Y., Yin, Z., Xue, X., and Zhou, B. (2014). An eight-miRNA signature as a potential biomarker for predicting survival in lung adenocarcinoma. *Journal of translational medicine* 12(1)**,** 159.

Lin, T., Fu, Y., Zhang, X., Gu, J., Ma, X., Miao, R., et al. (2018). A seven-long noncoding RNA signature predicts overall survival for patients with early stage non-small cell lung cancer. *Aging (Albany NY)* 10(9)**,** 2356.

Liu, G.M., Xie, W.X., Zhang, C.Y., and Xu, J.W. (2019). Identification of a four‐gene metabolic signature predicting overall survival for hepatocellular carcinoma. *Journal of cellular physiology* 235**,** 1624–1636.

Long, J., Zhang, L., Wan, X., Lin, J., Bai, Y., Xu, W., et al. (2018). A four‐gene‐based prognostic model predicts overall survival in patients with hepatocellular carcinoma. *Journal of cellular and molecular medicine* 22(12)**,** 5928-5938.

Makowsky, R., Pajewski, N.M., Klimentidis, Y.C., Vazquez, A.I., Duarte, C.W., Allison, D.B., et al. (2011). Beyond Missing Heritability: Prediction of Complex Traits. *PLoS Genetics* 7(4)**,** e1002051. doi: 10.1371/journal.pgen.1002051.

Mao, X., Qin, X., Li, L., Zhou, J., Zhou, M., Li, X., et al. (2018a). A 15-long non-coding RNA signature to improve prognosis prediction of cervical squamous cell carcinoma. *Gynecologic oncology* 149(1)**,** 181-187.

Mao, Y., Fu, Z., Zhang, Y., Dong, L., Zhang, Y., Zhang, Q., et al. (2018b). A seven-lncRNA signature predicts overall survival in esophageal squamous cell carcinoma. *Scientific reports* 8(1)**,** 8823.

Ott, J. (2015). Polygenic models for risk prediction in human genetics. *Human heredity* 80(4)**,** 162-164.

Ping, Z., Xiang, Z., and Huang, S. (2017). Prediction of gene expression with cis-SNPs using mixed models and regularization methods. *Bmc Genomics* 18(1)**,** 368.

Ripatti, S., and Palmgren, J. (2000). Estimation of multivariate frailty models using penalized partial likelihood. *Biometrics* 56(4)**,** 1016-1022.

Shen, S., Bai, J., Wei, Y., Wang, G., Li, Q., Zhang, R., et al. (2017a). A seven-gene prognostic signature for rapid determination of head and neck squamous cell carcinoma survival. *Oncology reports* 38(6)**,** 3403-3411.

Shen, S., Wang, G., Shi, Q., Zhang, R., Zhao, Y., Wei, Y., et al. (2017b). Seven-CpG-based prognostic signature coupled with gene expression predicts survival of oral squamous cell carcinoma. *Clinical Epigenetics* 9(1)**,** 88. doi: 10.1186/s13148-017-0392-9.

Shi, X.-H., Li, X., Zhang, H., He, R.-Z., Zhao, Y., Zhou, M., et al. (2018). A five-microRNA signature for survival prognosis in pancreatic adenocarcinoma based on TCGA data. *Scientific reports* 8(1)**,** 7638.

Speed, D., and Balding, D.J. (2019). SumHer better estimates the SNP heritability of complex traits from summary statistics. *Nature Genetics* 51(2)**,** 277-284. doi: 10.1038/s41588-018-0279-5.

Speed, D., Hemani, G., Johnson, M.R., and Balding, D.J. (2012). Improved heritability estimation from genome-wide SNPs. *The American Journal of Human Genetics* 91(6)**,** 1011-1021.

Therneau, T.M. (2019). coxme: Mixed Effects Cox Models. R package version 2.2-14. <https://CRAN.R-project.org/package=coxme>.

Therneau, T.M., Grambsch, P.M., and Pankratz, V.S. (2003). Penalized survival models and frailty. *Journal of computational and graphical statistics* 12(1)**,** 156-175.

Tibshirani, R. (1996a). Regression shrinkage and selection via the LASSO. *Journal of the Royal Statistical Society: Series B (Statistical Methodology)* 58(1)**,** 267-288.

Tibshirani, R. (1996b). Regression shrinkage and selection via the lasso. *Journal of the Royal Statistical Society: Series B (Methodological)* 58(1)**,** 267-288.

Tibshirani, R. (1997). The Lasso method for variable selection in the Cox model. *Statistics in Medicine* 16**,** 385-395.

Visscher, P.M., Hill, W.G., and Wray, N.R. (2008). Heritability in the genomics era—concepts and misconceptions. *Nature reviews genetics* 9(4)**,** 255.

Wu, H., and Zhang, J. (2018). Decreased expression of TFAP2B in endometrial cancer predicts poor prognosis: A study based on TCGA data. *Gynecologic oncology* 149(3)**,** 592-597.

Xiao, H., Xu, D., Chen, P., Zeng, G., Wang, X., and Zhang, X. (2018). Identification of five genes as a potential biomarker for predicting progress and prognosis in adrenocortical carcinoma. *Journal of Cancer* 9(23)**,** 4484.

Yang, J., Benyamin, B., McEvoy, B.P., Gordon, S., Henders, A.K., Nyholt, D.R., et al. (2010). Common SNPs explain a large proportion of the heritability for human height. *Nature genetics* 42(7)**,** 565.

Yang, J., Zeng, J., Goddard, M.E., Wray, N.R., and Visscher, P.M. (2017). Concepts, estimation and interpretation of SNP-based heritability. *Nature genetics* 49(9)**,** 1304.

Yazdi, M., Visscher, P., Ducrocq, V., and Thompson, R. (2002). Heritability, reliability of genetic evaluations and response to selection in proportional hazard models. *Journal of dairy science* 85(6)**,** 1563-1577.

Zeng, P., and Zhou, X. (2017). Non-parametric genetic prediction of complex traits with latent Dirichlet process regression models. *Nature Communications* 8(1).

Zhao, Q., Shi, X., Xie, Y., Huang, J., Shia, B., and Ma, S. (2014). Combining multidimensional genomic measurements for predicting cancer prognosis: observations from TCGA. *Briefings in bioinformatics* 16(2)**,** 291-303.

Zhao, X., Sun, S., Zeng, X., and Cui, L. (2018). Expression profiles analysis identifies a novel three-mRNA signature to predict overall survival in oral squamous cell carcinoma. *American journal of cancer research* 8(3)**,** 450.

Zhou, J., Wu, X., Li, G., Gao, X., Zhai, M., Chen, W., et al. (2017). Prediction of radiosensitive patients with gastric cancer by developing gene signature. *International journal of oncology* 51(4)**,** 1067-1076.

Zhou, X., Carbonetto, P., and Stephens, M. (2013). Polygenic modeling with Bayesian sparse linear mixed models. *PLoS Genetics* 9(2)**,** e1003264. doi: 10.1371/ journal.pgen.1003264.

Zhou, X., Wang, X., Huang, Z., Xu, L., Zhu, W., and Liu, P. (2014). An ER-associated miRNA signature predicts prognosis in ER-positive breast cancer. *Journal of Experimental & Clinical Cancer Research* 33(1)**,** 94.

Zou, H., and Hastie, T. (2005). Regularization and variable selection via the elastic net. *Journal of the royal statistical society: series B (statistical methodology)* 67(2)**,** 301-320.
